# CCAAT Promoter element regulates transgenerational expression of the MHC class I gene

**DOI:** 10.1007/s00412-024-00820-2

**Published:** 2024-06-26

**Authors:** Jocelyn D. Weissman, Aparna Kotekar, Zohar Barbash, Jie Mu, Dinah S. Singer

**Affiliations:** 1 Experimental Immunology Branch, Center for Cancer Research, National Cancer Institute, NIH, Bldg 10, Room 4B-36, Bethesda, MD 20892 USA; 2 Present Address: NIH Center for Human Immunology, Inflammation, and Autoimmunity (CHI), National Institute of Allergy and Infectious Diseases, NIH, Bethesda, MD 20892 USA; 3 Present Address: FORE Biotherapeutics, Philadelphia, PA USA

**Keywords:** Transgenerational inheritance, CCAAT box, Promoter element, MHC class I gene, Epigenetic modifications

## Abstract

Transgenerational gene expression depends on both underlying DNA sequences and epigenetic modifications. The latter, which can result in transmission of variegated gene expression patterns across multiple generations without DNA alterations, has been termed epigenetic inheritance and has been documented in plants, worms, flies and mammals. Whereas transcription factors binding to cognate DNA sequence elements regulate gene expression, the molecular basis for epigenetic inheritance has been linked to histone and DNA modifications and non-coding RNA. Here we report that mutation of the CCAAT box promoter element abrogates NF-Y binding and disrupts the stable transgenerational expression of an MHC class I transgene. Transgenic mice with a mutated CCAAT box in the MHC class I transgene display variegated expression of the transgene among littermates and progeny in multiple independently derived transgenic lines. After 4 generations, CCAAT mutant transgenic lines derived from a single founder stably displayed distinct patterns of expression. Histone modifications and RNA polymerase II binding correlate with expression of CCAAT mutant transgenic lines, whereas DNA methylation and nucleosome occupancy do not. Mutation of the CCAAT box also results in changes to CTCF binding and DNA looping patterns across the transgene that correlate with expression status. These studies identify the CCAAT promoter element as a regulator of stable transgenerational gene expression such that mutation of the CCAAT box results in variegated transgenerational inheritance. Considering that the CCAAT box is present in 30% of eukaryotic promoters, this study provides insights into how fidelity of gene expression patterns is maintained through multiple generations.

**Supplementary Information:**

The online version contains supplementary material available at 10.1007/s00412-024-00820-2.

## Introduction

Major histocompatibility complex class I genes are ubiquitously expressed, but their level of expression varies among tissues and is determined by both tissue-specific and hormonal signals. The MHC class I promoter consists of elements homologous to the canonical elements CCAAT, TATAA, Sp1 binding site (Sp1BS), and Initiator (Inr). The CCAAT element is completely conserved among MHC class I genes within a family and among mammalian species (Howcroft and Singer 2003). As we have reported previously, a transgene spanning the MHC class I gene, PD1, was expressed in mice with the same tissue-specific and cytokine-dependent patterns as the endogenous class I genes (Frels et al. 1985; Barbash et al. 2013). Surprisingly, as assessed by individually mutating each of the promoter elements in the context of the transgene, none were essential for class I expression. In particular, we found that the CCAAT box regulates constitutive expression of the MHC class I gene in a tissue-specific manner. Thus, mutation of the CCAAT box in the context of the MHC class I promoter does not affect constitutive expression in the spleen. However, it leads to significantly higher expression in kidney and brain indicating it functions as a repressor in non-lymphoid tissues (Barbash et al. 2013). These studies identified the CCAAT box as a tissue specific regulator of MHC class I transcription, functioning as an activator or repressor element in a context-specific fashion.

The CCAAT box is a common cis-acting element found in the promoters of about a third of eukaryotic genes. It is associated with the regulation of many housekeeping genes and genes involved in cellular processes such as growth and development. The presence of the CCAAT box enhances the efficiency of transcription initiation and contributes to the overall regulation of gene expression in eukaryotic cells. The CCAAT box is typically located upstream of the transcription start site and serves as a binding site for various transcription factors. The most common factor binding the CCAAT box is the trimeric complex, NF-Y, which consists of three subunits: NF-YA, NF-YB and NF-YC. Binding of NF-Y to the CCAAT box helps to stabilize the interaction of other transcription factors and RNA polymerase II with the promoter and facilitates the assembly of the transcription initiation complex. Importantly, NF-Y regulates expression of the major histocompatibility (MHC) class I and class II genes, as well as globin and albumin genes (Mantovani et al. 1992; Louis-Plence et al. 1997; van den Elsen 2011; Martyn et al. 2017). Consistent with the CCAAT box playing a critical role in transcriptional regulation, CCAAT box mutations are causative for a number of human diseases, including hemoglobinopathies (Fang et al. 2004).

In the present studies, we extend the characterization of the CCAAT box and report that it also functions to maintain stable MHC class I expression across generations. Mutation of the CCAAT transiently abrogates stable transgenerational patterns of expression. Among multiple independent CCAAT mutant (CCAATm) transgene founders, some founders expressed the transgene whereas others did not. However, in subsequent generations, progeny of non-expresser founders expressed the transgene. Those progenies, in turn, gave rise to transgenic progeny that were non-expressers. This generational alternation of expression occurred over multiple generations until stabilizing after a few generations, such that transgenic lines derived from a common founder displayed distinct patterns of expression. In contrast to the wild type CCAAT box, NF-Y does not bind the mutated CCAAT box. The CCAATm transgene expressers are distinguished from the non-expressers by their patterns of histone modification, Pol II and CTCF binding and DNA looping. These studies demonstrate that the conserved MHC class I CCAAT box functions both as a transcriptional regulator and to maintain stable expression; mutation of the CCAAT box leads to variegated expression between generations. We discuss the possible role of the CCAAT box in regulating transgenerational epigenetic inheritance.

## Results

### Mutation of the CCAAT promoter element of an MHC class I gene (PD1) results in alternating expression status between different generations

We have reported that a swine MHC class I transgene, PD1, is stably expressed over multiple generations, with patterns of expression that parallel those of the endogenous mouse MHC class I (Frels et al. 1985). To assess the role of core promoter elements in regulating expression of MHC class I genes, we had generated a series of transgenic mice with mutations in either the CCAAT box, the TATA box, Sp1 binding site (Sp1BS) or INR of class I gene, PD1 (Barbash et al. 2013). In that study, we found that all mutant promoters were capable of supporting expression of the class I transgene. Upon further breeding of these transgenic mice, we were surprised to find that whereas the TATA box, Sp1BS and INR mutant transgenes all displayed stable transgenerational expression, the CCAAT box mutant displayed variegated expression of the transgene among different founders and their progeny.

**Fig. 1:**
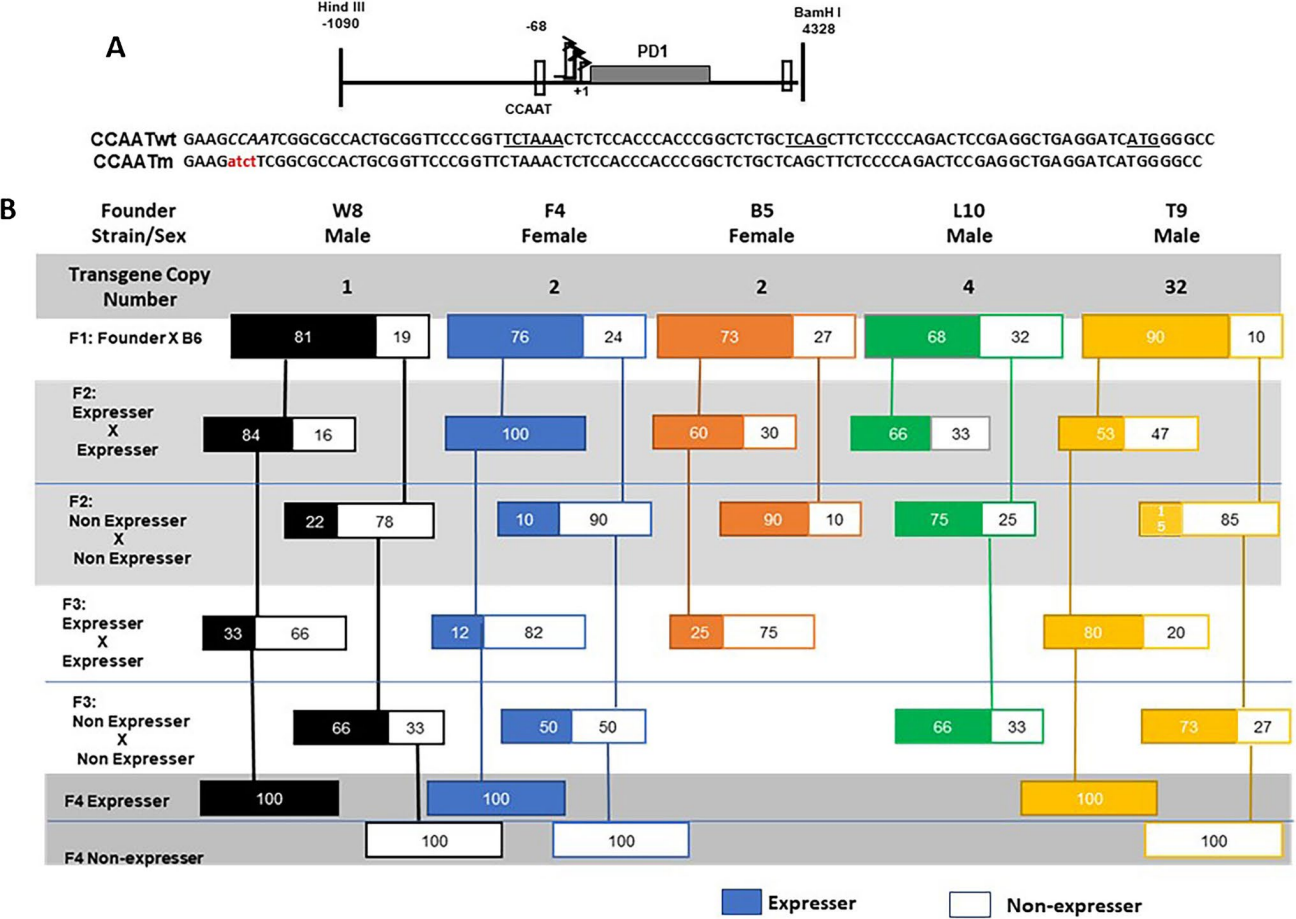
Mutation of the CCAAT box results in variable gene expression across generations. **A** Both CCAATwt and CCAATm transgenes contain the same backbone DNA segment containing 1kb of 5’ flanking sequences, 3.3kb of MHC class I transgene, PD1, coding sequences (gray rectangle) and 1kb of 3’ sequences. The mutation introduced into the CCAAT sequence (italicized) is indicated in red; the CCAATwt and mutant CCAAT sequences within the proximal promoter are centered at -68 bp, relative to the major TSS at + 1. Transcription of both CCAATwt and CCAAT mutant promoters initiate at the same sites, with a major site at + 1. The positions of the TATAA-like element at -30 bp, the Inr at + 1, and ATG translation start site are underlined. **B** Variegated expression of MHC class I transgene, PD1, across multiple generations of transgenic mice with a mutated CCAAT core promoter element. The generational inheritance patterns of 5 different founder mice with the transgene copy number and sex of the founders indicated. Transgene-positive off-spring were analyzed by FACS for cell surface PD1 expression on PBL. Only transgene-positive mice that express (colored solid boxes) or do not express (outlined, white boxes) are shown

Of 32 independently derived transgenic founder mice with CCAAT box mutations (Fig. 1A), 28 founder mice expressed the CCAAT mutant transgene at levels equal to or greater than mice with the wild type CCAAT box (CCAATwt) transgene; the remaining 4 failed to express the PD1 transgene. Five CCAAT mutant founders that expressed the transgene were bred to establish independent, homozygous lines of CCAAT mutant (CCAATm) transgenes (Fig. 1B). Pups were monitored for the presence of the PD1 transgene by PCR and for expression of the PD1 protein on peripheral blood lymphocytes (PBL) by flow cytometry. All 5 founders that expressed the transgene gave rise to offspring that also expressed the transgene (Fig. 1B). Of note, all 5 lines also generated offspring that carried the transgene but did not express PD1 on their PBL. In subsequent generations of *inter se* breeding, CCAATm mice expressing the PD1 transgene gave rise to offspring that were genotype positive for the transgene and expressed the gene while others were also genotype positive but did not express the transgene (Fig. 1B). Significantly, genotype-positive non-expressers generated genotype-positive offspring that expressed the transgene as well as others that did not, independent of sex (Fig. 1B, Fig. 1S). All transgene promoter regions were re-sequenced to verify that both expresser and non-expresser transgenes had mutated CCAAT boxes. Additionally, level of expression in CCAATm expressers was consistent across generations regardless of the expression pattern of the parents, and the expression status/level did not change over the lifetime of an individual mouse.

Thus, expression of the CCAAT mutant transgene flipped between generations.

This pattern of variegated expression continued in each independently derived line for multiple generations after which the phenotype was stable and remained stable in all subsequent generations. The variegated expression is unlikely due to insertion site effects, since it was observed in 5 independently derived founders. It is also unlikely due to copy number effects, since three of the lines had low (1–2) copy numbers (Fig. 1B). Transgenerational variegation of transgene expression was restricted to the CCAATm mice and was not observed in any of the 48 lines of PD1 mice where the CCAAT box was not mutated (these transgenic lines included mice with CCAATwt transgene as well as with other core promoter mutants i.e. in the TATA box, SP1BS or INR mutants) (Barbash et al. 2013).

**Fig. 2:**
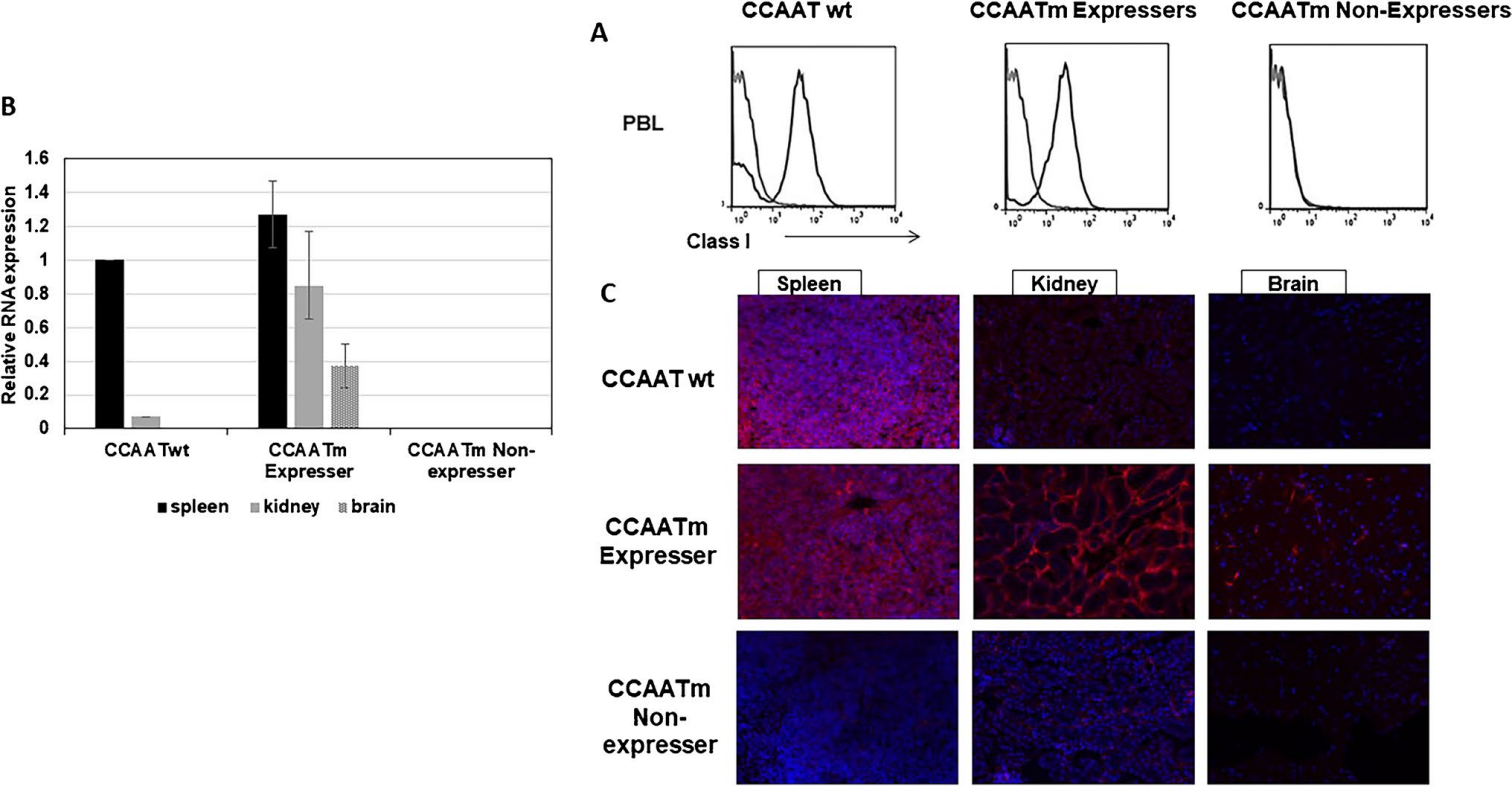
CAATm transgenic mice differ in their MHC class I expression in multiple tissues. **A** Representative FACS profiles of PBL of the three transgenic lines. PBL were stained with an anti-PD1 antibody (black profile); secondary antibody alone served as the negative control (gray profile). Data are representative of > 3 independent experiments. **B** Real-time qPCR was performed on total RNA extracted from tissues of CCAATwt and both CCAAT mutant transgenic lines. The levels of RNA were normalized to 18S RNA in each tissue. Data are average ± SEM from 3 individuals of each line. RNA levels in the tissues were all normalized to the RNA level in spleen of CCAATwt mice which was set to 1. **C** Frozen sections of spleen, kidney and brain from CCAATwt, CCAATm expresser and CCAATm non-expresser transgenic mice were immunostained with anti-PD1 antibody and fluorescent goat anti-mouse Ig. Slides were counterstained with DAPI. Images are representative of two independent experiments

All further investigations were done in mice that had fixed their expression status as either expressers or non-expressers i.e., after the 4th generation. As shown previously (Barbash et al. 2013), the CCAATm mice that stably express PD1 do so in all tissues analyzed. Thus, CCAATm transgenic mice that expressed the PD1 protein on their PBL (Fig. 2A) also expressed RNA and protein in their tissues (i.e., spleen, kidney, brain) (Fig. 2B, C). As also described previously (Barbash et al. 2013), expression of the PD1 transgene in the kidney and brain of CCAATm mice is significantly higher than in CCAATwt mice indicating that the CCAAT box regulates tissue-specific transcription (Fig. 2B, C). Specifically, the CCAAT box appears to negatively regulate PD1 transcription in some non-lymphoid tissues. CCAATm non-expressers that did not express PD1 protein on their PBL, did not express either PD1 protein or RNA in their spleen, kidney or brain (Fig. 2A-C).

We next considered the possibility that the CCAATm transgenic non-expressers could be induced to express the PD1 transgene in response to external stimuli, such as interferon. As expected, *in vivo* γ-interferon treatment increased the levels of PD1 RNA in the tissues of both CCAATwt and CCAATm expressers (Fig. 2SA). Importantly, it did not induce *de novo* expression of PD1 in the non-expresser CCAATm transgenic mice, although it did enhance endogenous H2-K^b^ expression in these mice (Fig. 2SB). Furthermore, mating of non-expresser CCAATm mice, either male or female, to C57BL/6 mice did not induce expression in the progeny. Therefore, the lack of PD1 expression by the CCAATm non-expresser mice is an intrinsic defect caused by the CCAAT mutation.

**Fig. 3:**
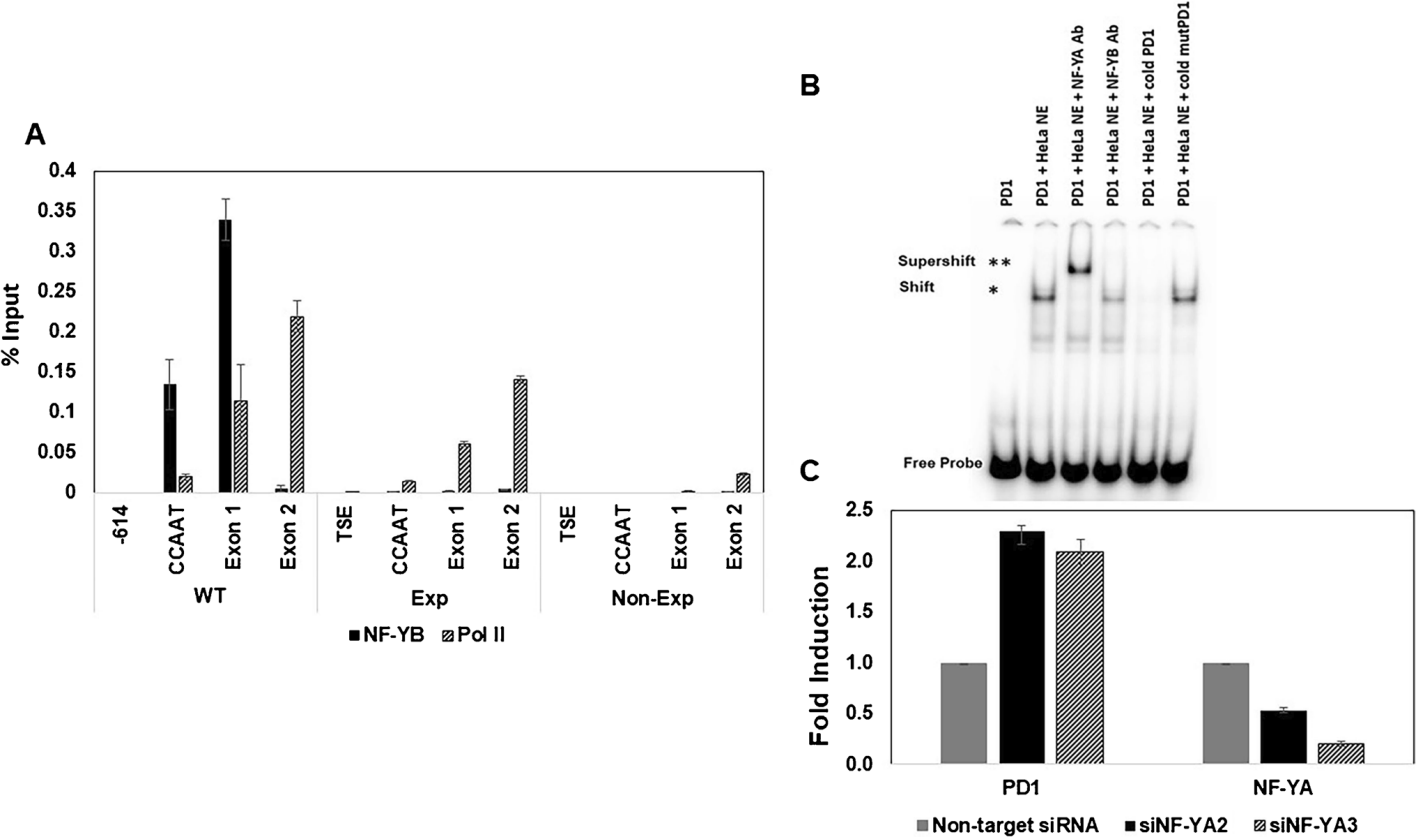
NF-Y binds PD1 CCAAT box both *in vitro* and *in vivo,* and regulates MHC class I expression. **A** ChIP analysis of NF-YB binding to chromatin from spleens of CCAATwt, CCAATm expresser and CCAATm non-expresser transgenic strains. Results are expressed as % of total Input and error bars represent standard error of mean (SEM) of 2 technical replicates. Note: X axis demarcates location relative to the TSS and is not to scale. Data are representative of 2 independent experiments. Pol II ChIP served as positive control where binding was seen in CCAATwt as well as CCAATm expressers but not in CCAATm non-expressers. **B** Gel shifts using a radiolabeled PD1 CCAATwt or CCAATm DNA fragment (1011–1060 bp) with or without HeLa nuclear extracts, in the presence or absence of NF-Y antibodies or 1000-fold excess of unlabeled double-stranded CCAATwt or CCAATm oligonucleotide competitors as indicated. **C** Quantitation of fold-induction of PD1 gene expression on siRNA knockdown of NF-YA in mouse L cells stably transfected with PD1 (93B2 cell line; NF-YA2 and NF-YA3 were 2 siRNAs targeting NF-YA). siRNA knockdowns at 24 h are shown. The bars labeled PD1 show PD1 RNA levels in cells treated with NF-Y siRNA, expressed relative to the PD1 levels in non-targeting siRNA treated cells set to 1. The bars labeled NF-Y show knock-down of NF-Y RNA levels in NF-Y siRNA treated cells versus cells treated with non-targeting siRNA set to 1. For both PD1 and NF-Y RNA, 18S RNA was used as internal control and error bars represent standard error of mean (SEM) of 2 technical replicates. The experiment is representative of two independent experiments

Taken together, these findings demonstrate that mutation of the CCAAT box leads to variegated transgenerational expression of the PD1 transgene across generations, where expression status of offspring may vary from that of the parents. Over time, the expression status stabilizes and thereafter the CCAATm offspring are stable expressers or non-expressers. To confirm the role of the CCAAT element in maintaining stable transgenerational expression, a second, independent set of PD1 transgenic mice was established with the same CCAAT mutation. These CCAATm transgenic mice displayed the same transgenerational flipping of expression where expressers gave rise to both expressing and non-expressing offspring and vice-versa (Fig. 3S).

From these results we conclude that the MHC class I CCAAT element has a dual function- as a regulator of transcription and as a determinant of stable transgenerational epigenetic inheritance.

### NF-Y regulates MHC class I expression through the CCAAT box

CCAAT box promoter elements are known to be bound by the trimeric NF-Y complex consisting of NF-YA, NF-YB and NF-YC (Maity et al. 1992; Bi et al. 1997). The PD1 CCAAT box, centered at -68 bp upstream of the transcription start site (TSS), matches the NF-Y binding consensus sequence. To determine whether NF-Y binds to the PD1 CCAAT box, we first performed ChIP-PCR on chromatin from spleen with NF-YB antibody (Fig. 3A). Whereas NF-YB antibody immunoprecipitated chromatin derived from a region spanning the CCAAT box and exon 1 of the CCAATwt, no immunoprecipitation was observed with chromatin from either CCAATm expresser or non-expresser. These results indicate that NF-Y binds to the wild type, but not mutant, CCAAT box. To confirm that NF-Y binds to the CCAATwt but not to CCAATm transgene, we also performed an Electrophoretic Mobility Shift Assay (EMSA) using a 50 bp fragment of the PD1 promoter spanning the CCAAT box (Fig. 3B). Incubation of the PD1 promoter fragment with HeLa nuclear extract resulted in an electrophoretic mobility shift, which was supershifted by the addition of anti-NF-YA antibody, indicating that the complex contains NF-YA; NF-YB antibody reduced the intensity of the shifted band, suggesting competition for binding the NF-Y. 1000X excess of unlabeled (cold) CCAATwt probe, but not a CCAATm probe, competed away the complex. These results demonstrate the specific binding of NF-Y to the PD1 WT CCAAT box *in vitro*.

As compared to CCAATwt transgenic mice, CCAATm expresser mice express higher levels of PD1 RNA and protein in non-lymphoid tissues like kidney and brain (Fig. 2B and C). The finding that NF-Y binds *in vitro* and *in vivo* to the CCAATwt but not to the mutant CCAAT box, suggested that the increased expression resulted from the absence of NF-Y binding to the mutant CCAAT box *in vivo*. To test this hypothesis, NF-Y was knocked-down in a mouse fibroblast L-cell line stably transfected with the CCAATwt PD1 gene using two siRNAs targeting NF-YA (Fig. 3C). As predicted, depletion of NF-YA by either siRNA resulted in increased levels of PD1 transcripts, relative to the non-targeting siRNA control (Fig. 3C). These findings are consistent with the aberrantly high expression of the CCAATm transgene in the non-lymphoid cells of CCAATm expressers and demonstrate that NF-Y functions as a negative transcriptional regulator of MHC class I expression in non-lymphoid cells.

### Pol II occupancy correlates with CCAATm transgene expression status

**Fig. 4:**
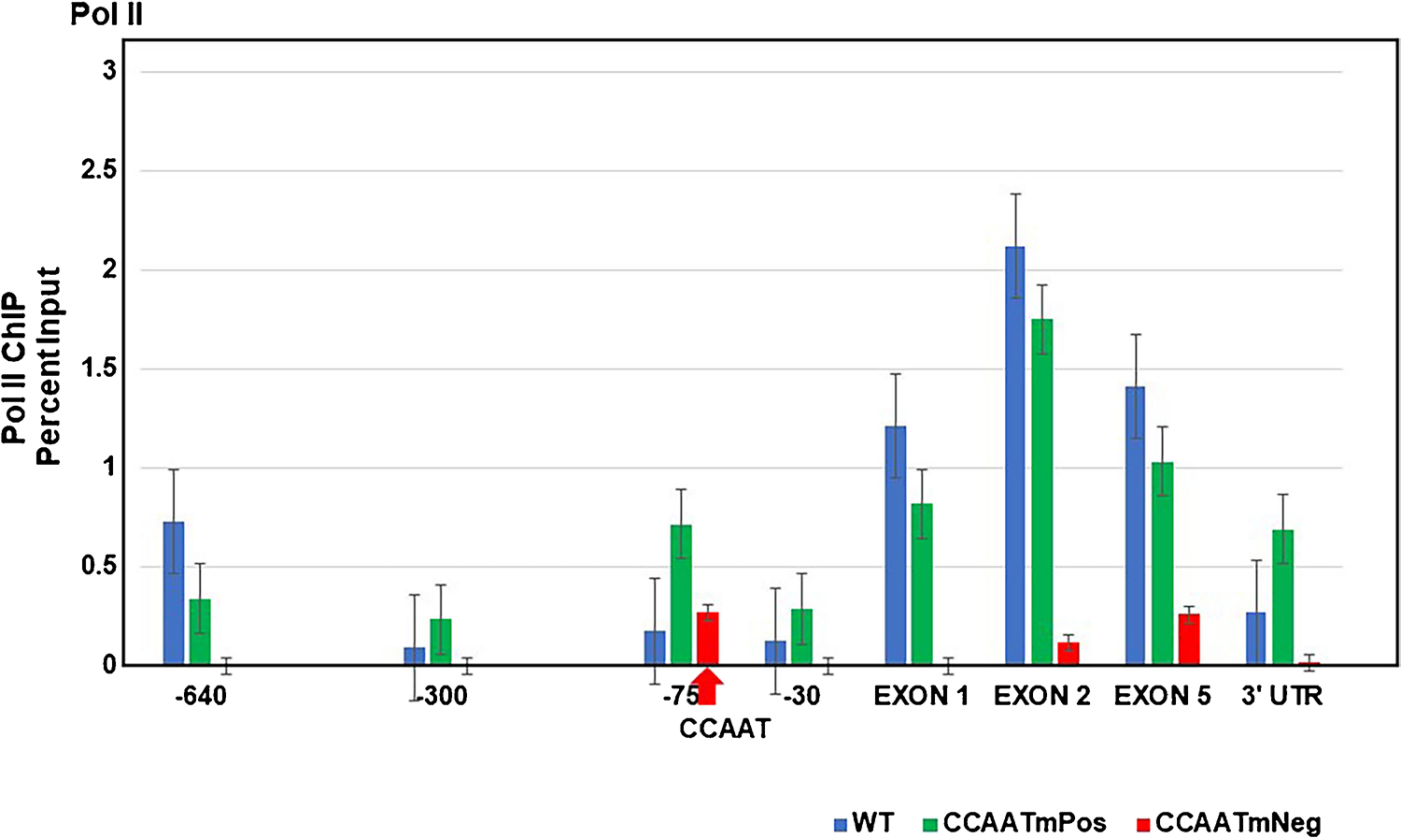
Pol II differs in its association with the CCAATm expresser and non-expresser transgenes. ChIP analysis of Pol II binding to chromatin from spleens of CCAATwt, CCAATm expresser and CCAATm non-expresser transgenic strains. Results are expressed as % of total Input. Note: X axis demarcates location relative to the TSS and is not to scale. Location of CCAAT box is denoted by arrow. Data represent 3 independent experiments

To begin to investigate the basis of transgenerational heterogeneity of expression of PD1 in the CCAAT mutant lines, we first examined Pol II occupancy across the PD1 transgene in splenocytes of CCAATm expressers and non-expressers, as well as CCAATwt transgenic mice. ChIP analysis showed Pol II occupancy at the promoter and across the transgene in both CCAATwt and CCAATm expresser mice (Fig. 4). Overall, the levels of Pol II occupancy across the transgene were comparable between CCAATwt and the CCAATm expressers, except around -75 bp where Pol II levels were markedly higher in CCAATm expressers as compared to CCAATwt mice*.* In contrast, no significant Pol II was detected across the body of the PD1 gene of the non-expresser transgene, consistent with the lack of transcription. Surprisingly, Pol II was detected at -75bp of the non-expresser transgene. This suggests that in both expresser and non-expresser transgenic lines, Pol II loads at -75 but is only able to progress in the expresser (Fig. 4).

Pol II occupancy was also detected at distal upstream regulatory regions of the CCAATm expresser, but not the non-expresser, consistent with our observations that the upstream regulatory regions of the PD1 gene are transcribed in PD1 expressing mice but not in the non-expressers (Fig. 4S).

Thus, Pol II occupancy at the PD1 gene reflects the expression status of the gene.

### Histone modifications correlate with expression status of the PD1 gene

**Fig. 5:**
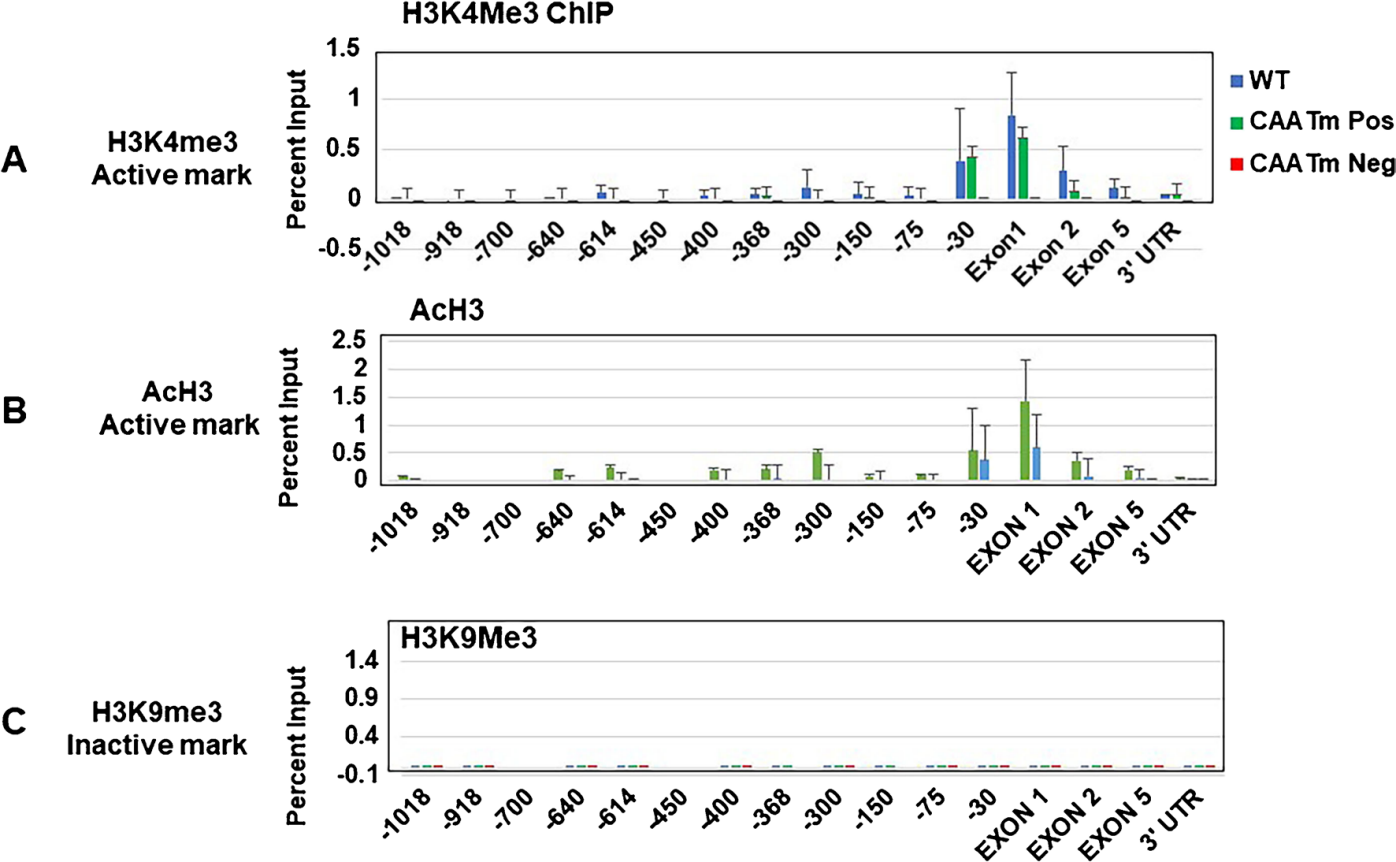
Histone marks of active genes occur only on CCAATwt and CCAATm expresser transgenes. ChIP analysis of AcH3K4me3 (**A**), AcH3 (**B**), and H3K9me3 (**C**) on chromatin from spleens of CCAATwt, expresser, and non-expresser CCAATm transgenic strains. Results are presented as % of total Input. Note: X axis demarcates location relative to the TSS and is not to scale. Data are representative of 3 independent experiments

**Fig. 6:**
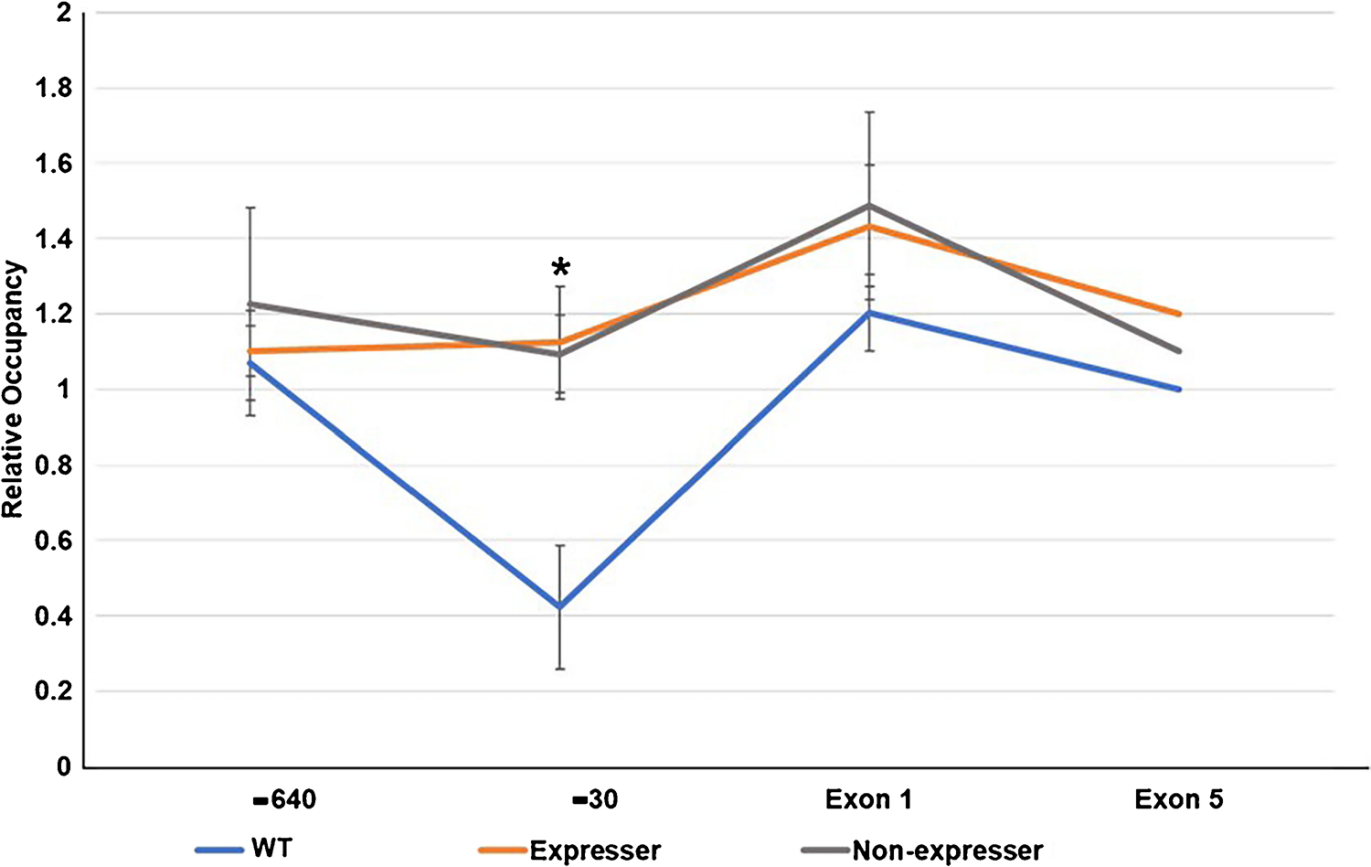
Nucleosome occupancy is higher in both CCAATm transgene lines than in the CCAATwt transgene. Nucleosome occupancy across CCAATm transgene of stable expressers and non-expressers and CCAATwt in spleen. The data include the levels of occupancy at -640, -30, exon 1 and exon 5. The Y axis displays the nucleosomal occupancy following chromatin treatment with 3–5 units MNase relative to the undigested control. Data are the average ± SEM of 2 independent experiments at two concentrations. Asterisk denotes a p value ≤ 0.047

We next asked whether the differences in Pol II occupancy and expression status of the CCAATm transgenic mice result from differences in chromatin organization or structure. As histone modifications are correlated with epigenetic inheritance, we analyzed the levels of chromatin marks that influence chromatin structure and gene expression. We assessed both histone marks associated with open chromatin and actively transcribing genes (e.g. histone H3 acetylation (H3Ac); histone H3 lysine 4 trimethylation (H3K4me3)) and repressive histone chromatin marks (e.g. histone H3 lysine 9 trimethylation (H3K9me3) and H3 lysine 27 trimethylation (H3K27me3)), as well as DNA methylation, in spleen cells of the CCAATm and CCAATwt transgenic mice (Fig. 5, 5S and 6S).

In splenocytes of CCAATwt and CCAATm expresser mice, the PD1 promoter proximal region and gene body were associated with active histone marks, H3Ac and H3K4me3, across the PD1 transgene (Fig. 5A and B). In sharp contrast, the CCAATm non-expresser transgenic mice lacked these positive histone modifications across the PD1 gene. Interestingly, the repressive histone mark H3K9Me3 was not observed to any significant level across the PD1 transgene in any of these mice, including non-expressers (Fig. 5C); H3K27me3 also did not correlate with expression status (Fig. 5S). Additionally, DNA methylation patterns did not differ markedly between expressers and non-expressers, although both patterns differed from the CCAATwt transgene (Fig. 6S).

**Fig. 7:**
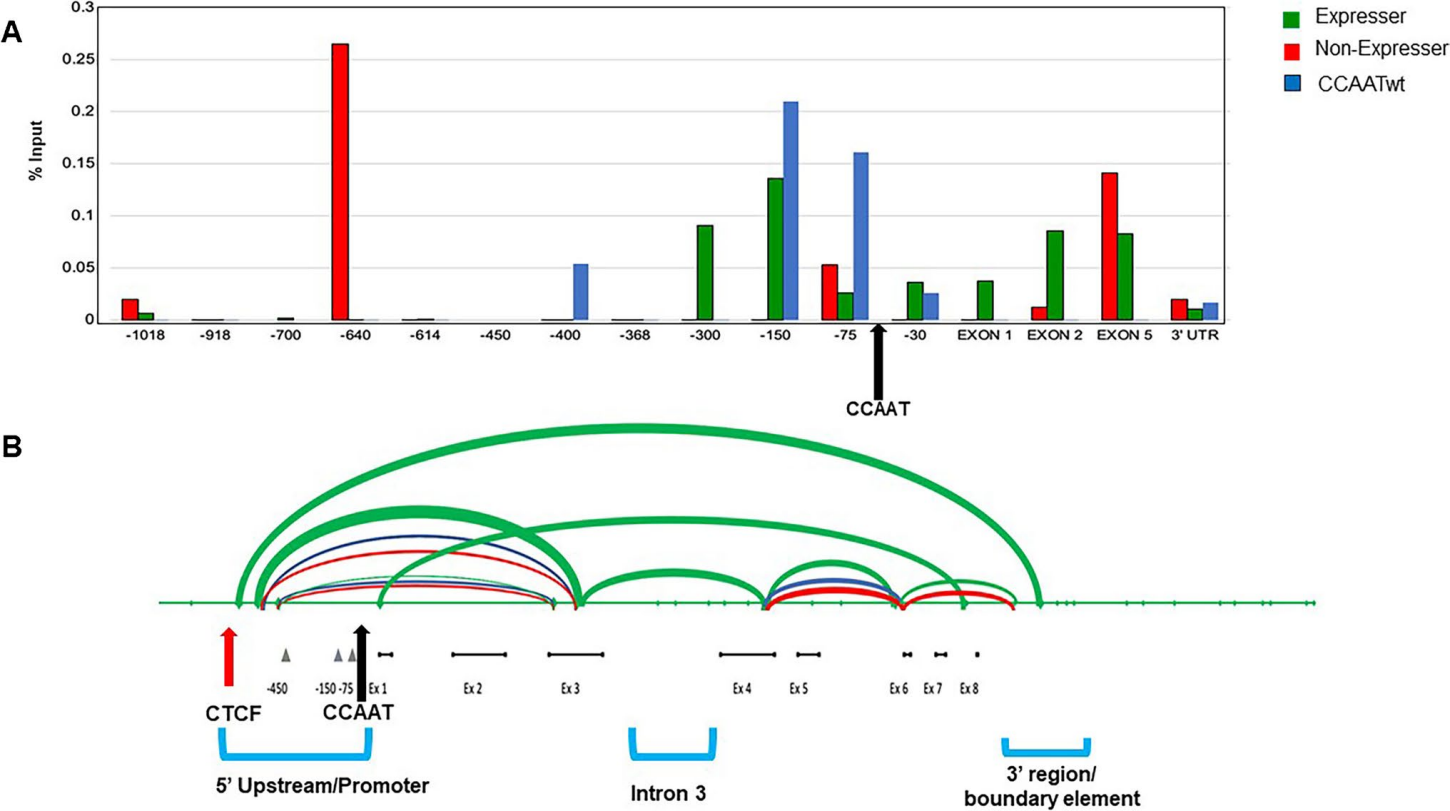
CCAATm transgenes show distinct patterns of CTCF binding and DNA looping. **A** ChIP analysis of CTCF binding on chromatin from spleens of CCAATwt, expresser and non-expresser CCAATm transgenic strains. Results are presented as % of total input and are representative of 3 independent experiments. Note: X axis denotes location relative to the TSS and is not to scale. Arrow indicates position of the CCAAT box. **B** CCAATm expresser transgenes generate DNA loops not seen in either CCAATwt or the CCAATm non-expressers transgenes. The endpoints for each loop are located at sites of enzymatic digestion which were ligated as seen by flanking PCRs using the oligos listed in Methods. The data are representative of at least 3 independent experiments. The thickness of the loop correlates with intensity of the PCR bands. The location of the CCAAT box is denoted by a black arrow. The 5’ Upstream/Promoter, Intron 3, and 3’ region/boundary element are indicated. The region of CTCF binding in the CCAATm non-expresser is indicated by a red arrow

In the independently derived second set of CCAATm mice (Fig. 3S), splenic chromatin from CCAATm expresser mice was modified with H3Ac and H3K4me3, but not H3K9me3 (Fig. 7S), as was the original set of CCAATm mice. Chromatin from the CCAATm non-expresser splenocytes was not modified with either H3Ac and H3K4me3, as observed with the original set, but was modified with H3K9me3 marks. The basis for the difference in H3K9me3 pattern between the two sets of mice is not known but suggests that transgenerational transgene expression is not determined by the presence or absence of repressive histone marks.

## Nucleosome occupancy does not correlate with PD1 expression status

We reported previously that the CCAATwt PD1 promoter region is nucleosome-free (Kotekar et al. 2008), consistent with expression of the transgene. Therefore, we next considered the possibility that the differential expression in CCAATm mice reflected differences in nucleosome occupancy. Nucleosome occupancy across the PD1 transgene was assessed by limiting MNase digestion of nuclei prepared from splenocytes of CCAATwt, CCAATm expressers and non-expressers; DNA recovery was determined by real time PCR. As shown previously, chromatin around the CCAATwt promoter was hypersensitive to digestion, indicating a relative paucity of nucleosomes across this region (Fig. 6). Consistent with the lack of PD1 transcription in the CCAATm non-expresser mice, nucleosome occupancy was much higher across the PD1 promoter. However, unexpectedly, nucleosome occupancy was also high across the PD1 promoter of the CCAATm expresser mice. This finding of similarly high nucleosome occupancy in the expresser transgene was surprising, given its expression status.

To examine the possibility that there are subtle differences in nucleosome organization between the CCAATm expressers and non-expressers, chromatin from each was subjected to extensive MNase digestion. The relative susceptibility to digestion of the transgene – a measure of nucleosome occupancy—was assessed by determining DNA recovery in both supernatant (MNase-susceptible) and pellet (MNase-resistant) fractions (Fig. 8S). Under conditions of high MNase digestion, the promoters of both CCAATm transgenes were relatively inaccessible compared to the CCAATwt promoter. However, the promoter of the CCAATm expresser transgene was modestly more accessible than the non-expresser, suggesting that this small difference may be sufficient for Pol II loading and elongation.

Thus, although there are small differences in nucleosome occupancy between the CCAATm promoters of expressers and non-expressers, the overall nucleosomal occupancy at the CCAATm promoter does not correlate with expression.

### CCAATm non-expresser mice display a novel pattern of CTCF binding

The small difference in chromatin accessibility observed at the promoters of CCAATm expressers and non-expressers might explain in part their patterns of expression. However, it is likely that some other mechanism establishes and maintains their differential expression. We have previously reported an insulator element associated with the PD1 transgene, located 3’ to the polyA addition site, that is necessary for stable transgene expression (Cohen et al. 2009). We thus considered the possibility that mutation of the CCAAT box altered insulator function associated with the PD1 gene. Since insulator function is often mediated by binding of CTCF (Cuddapah et al. 2009), we next examined the patterns of CTCF binding to the CCAATwt and CCAAT mutant transgenes in splenic chromatin by ChIP analysis. CTCF binding across the CCAATwt PD1 transgene was variable primarily centered around the promoter proximal region and gene body (Fig. 7A. Figure 9S). CTCF also bound in the same regions in the CCAATm expresser proximal to the promoter. CTCF binding was not detected in the distal promoter region of either the CCAATwt or CCAATm expresser. In sharp contrast, in the CCAATm non-expresser, CTCF binding was consistently observed at the 5’ distal promoter; variable CTCF binding was observed in the promoter proximal region (Fig. 7, Fig. 9S). CTCF also bound within the gene body around exon 5 of the CCAATm non-expresser. The promoter distal, but not proximal, binding of CTCF correlates with lack of expression in the CCAATm non-expresser mice. The differential binding of CTCF at the 5’ distal site appears to be independent of methylation, since methylation is not detected in this region in any of the mice (Fig. 6S).

Thus, the CCAATm non-expressers are distinguished from the expressers and WT by their CTCF binding patterns.

### Distinct DNA looping patterns correlate with PD1 gene expression in CCAATm mice

CTCF is known to mediate both long and short loop formation through dimerization of two CTCF molecules bound at distal sites. Long loop formation is facilitated by cohesin, whereas short loop formation often occurs independently of cohesin and brings together promoter and gene body regions (Merkenschlager and Odom 2013; Ruiz-Velasco et al. 2017). The novel upstream binding of CTCF to the non-expresser transgene, in combination with its binding to the 3’ region of the gene, raised the possibility that CTCF forms novel chromatin loops in the non-expresser transgene. Therefore, we examined whether local looping occurs across the PD1 transgene by 3C chromosome capture (Fig. 7B, Fig. 10S). All 3 transgenes shared two major loops. One loop is centered around the promoter and spans the CCAAT box. Its formation is independent of NF-Y binding since both the WT and CCAATm transgenes form this loop. It also should be noted that the CTCF binding site in the CCAATm non-expresser lies outside of the loop that maps to the 5’ promoter region. Thus, CTCF binding is unlikely to affect formation of this loop. The second common loop is centered between exon 5 and exon 6. CTCF binds to this region in both the CCAATm expresser and non-expresser but not the WT, making it unlikely that it is involved in regulating either loop formation or expression.

Surprisingly, the CCAATm expresser formed an additional loop that is centered around intron 3 and not found in either the CCAATwt or CCAATm non-expresser transgenes (Fig. 7B). What factors contribute to the formation of this loop in the CCAATm expresser remain to be determined. However, these findings suggest that, in the absence of the wild type CCAAT box, the unique loop in the CCAATm expresser gene serves to protect the gene from inactivation.

## Discussion

In the present study, we have discovered that the CCAAT box associated with the MHC class I promoter is a regulator of stable transgenerational gene expression.

Mutation of the CCAAT box results in the failure of NF-Y binding, aberrant patterns of MHC class I expression and the transgenerational variegation of expression of the MHC class I transgene. Thus, the MHC class I CCAAT box functions as both a regulator of transcription and to maintain stable gene expression across generations. These findings lead to the hypothesis that the CCAAT box is a regulator of transgenerational epigenetic inheritance.

Transgenerational epigenetic inheritance (TEI) is the process by which novel phenotypes are transmitted from one generation to the next through epigenetic, not DNA sequence, changes (Cavalli and Heard 2019; Fitz-James and Cavalli 2022). While it has been widely documented in plants, the number of examples of TEI in mammals is still limited but increasing (Arzate-Mejia and Mansuy 2022). Although the mechanisms underlying TEI are not known, a number of epigenetic associations have been established in both plants and mammals. Among the documented links with epigenetic inheritance are changes in DNA methylation, small RNAs and histone modifications (Cavalli and Heard 2019; Burton and Greer 2022; Fitz-James and Cavalli 2022). Among the CCAAT mutant transgenic mice, MHC class I expression status did not correlate with patterns of DNA methylation across the transgene. Similarly, we observed only minor differences in nucleosomal packaging between expressers and non-expressers. Thus, these two potential mechanisms are not operational for TEI associated with the CCAAT box mutant. In contrast, expression of the transgene did correlate with histone modifications. Namely, the presence of H3K4me3 and total H3 acetylation was associated with the CCAATm expresser transgenic mice, but not the non-expressers.

Given the large-scale epigenetic reprograming in mammals during embryogenesis, other mechanisms—such as non-coding RNAs, 3D genome organization, and transcription factor binding—have been proposed to contribute to mammalian TEI (Fitz-James and Cavalli 2022). Indeed, all three of these features distinguish the CCAATm expresser from the non-expresser. Whereas the CCAATm expresser transgene transcribes promoter-distal non-coding RNA sequences, the CCAATm non-expresser does not. However, whether this is the mechanism determining TEI or a reflection of the ability of Pol II to initiate transcription cannot be distinguished. Significantly, the CCAATm expressers and non-expressers differ both in their 3D genome organization and transcription factor binding. The CCAATm expresser forms a unique 3D loop not found in either the CCAATm non-expresser or the WT transgene. This loop encompasses exon 3, intron 3 and exon 4. The role of this loop in expression of the CCAATm transgene, as well as its precise anchors, remains to be determined. Conversely, CTCF binds to a region in the 5’ distal promoter of the CCAATm non-expresser, but not to the CCAATm expresser. We speculate that CTCF binding to this region acts as a barrier to expression, perhaps by loop formation with the previously-identified 3’ barrier element (Cohen et al. 2009), although we have not been able to detect such a loop to date. Thus, there is a reciprocal relationship between CTCF binding and loop formation that correlates with expression; whether the establishment of one prevents the other remains to be determined. These findings extended previous reports that allele-specific differences in CTCF binding can lead to altered chromatin environment, 3D structure and gene expression (McDaniell et al. 2010). The variegated patterns of PD1 expression that occur in the early CCAATm transgene generations reflect stochastic establishment of either loops or CTCF binding. What leads to a stable, heritable configuration after the early generations remains to be determined.

The present studies suggest that the CCAAT box plays a singular role in the stable expression of the MHC class I gene. Thus, none of the forty-eight transgenic lines generated from either the WT MHC class I gene or other MHC class I promoter mutants (mutants in the TATA box, SP1 BS or Inr) displayed the variegated expression observed in the multiple CCAATm transgenic lines. There is only limited previous evidence for the role of a DNA sequence element in regulating transgenerational inheritance. DNA methylation of CpG islands associated with promoters is prevented by the presence of a promoter-associated Sp1-binding site; whether Sp1 is the responsible transcription factor was not directly demonstrated (Brandeis et al. 1994). In the present study, mutation of the CCAAT box abrogates NF-Y binding, further suggesting that NF-Y plays a critical role in maintaining stable expression. We speculate that the loss of stable transgenerational inheritance of the phenotype upon mutation of the CCAAT box results from loss of NF-Y binding leading to aberrant looping and transcription factor binding. In wild-type animals, NF-Y binding presumably establishes a favorable 3D chromatin structure that results in stable expression of the Class I gene across generations. NF-Y has been shown to contribute to zygotic genome activation and formation of DNase hypersensitive sites at the 2-cell stage (Lu et al. 2016). So, loss of NF-Y binding in CCAAT mutants presumably results in stochastic expression very early in embryogenesis leading to differential expression in littermates. This expression status is maintained throughout the lifespan of the individual but is reprogrammed and reset in subsequent generations.

As Rothi and Greer (Rothi and Greer 2023) pointed out in a recent review, the understanding of TEI now needs to go beyond correlation to causation, about which relatively little is known. Recent studies in C. elegans have begun to track heritable molecules that transmit epigenetic information. In worms, viral infections and starvation result in transgenerational inheritance of small RNAs that influence the transcriptome (Rechavi et al. 2011, 2014). Unlike previous examples of transgenerational inheritance which result from environmental triggers, these studies define a novel mechanism of transgenerational inheritance caused by a discrete mutation within a transcription factor binding site, leading to formation of novel DNA loops. Although the mechanisms underlying the distinct patterns of expression remain to be determined, our demonstration, in multiple independent transgenic lines, that mutation of the CCAAT box results in transgenerational inheritance provides a clear example of causation in mammals.

### Material and methods

#### Mice

 C57BL/10 mice homozygous for the MHC class I transgene PD1 (CCAATwt) were generated as described previously (Frels et al. 1985). The CCAATwt transgene contains a 1 Kb regulatory region upstream of the TSS, the entire coding region and 650bp immediately following the polyadenylation site that contains the 3’ boundary element (Cohen et al. 2009). Experiments were performed on CCAATm expresser and non-expresser lines derived from at least two independent founders (Barbash et al. 2013). Peripheral blood lymphocytes (PBL) were analyzed by flow cytometry of cells stained with anti-class I antibody. All animal procedures reported in this study that were performed by NCI-CCR affiliated staff were approved by the NCI Animal Care and Use Committee (ACUC) and in accordance with federal regulatory requirements and standards. All components of the intramural NIH ACU program are accredited by AAALAC International.

#### IFN treatment

Mice were injected intraperitoneally with 50kU of mouse IFNγ (CalBiochem) or an equal volume of PBS + 0.1%BSA. Tissues were harvested 24 h post-injection. Levels of PD1 and endogenous H2-K^b^ RNA were assessed by RT real time PCR of RNA extracted from tissues (RT-PCR described below). The experiment was performed twice each with three mice that were analyzed independently.

#### Flow cytometry

FACS was carried out using antibodies for PD1 surface expression (PT85, VRB) as described before (Ehrlich et al. 1989). FACS results were analyzed using FlowJo (BD).

#### Immunofluorescence

Immunofluorescence staining was performed according to standard protocol, using the PT85 antibody that was used for FACS staining. Slides were counterstained with DAPI.

#### Electrophoretic Mobility Shift Assay (EMSA)

NF-Y EMSA was done as described previously (Weissman and Singer 1991) with modifications mentioned below. DNA fragments used in the gel shift assay were the PD1 fragment from 1011–1060 and the corresponding CCAAT mutant fragment (IDT). DNA fragments were end labeled with [32P]ATP using T4-Polynucleotide kinase (NEB). EMSAs were done with 3.6ug of HeLa nuclear extract and 1.5-fmol radiolabeled probe in binding buffer (20mMTris-HCl pH 7.5, 50 mM NaCl, 5 mM MgCl2, 0.5 mM EDTA, 6.5% glycerol, 2.5 mM DTT, and 0.1 mg/ml BSA) (Bernardini et al. 2019) and 2ug poly(dA-dT). Reactions were incubated at 30°C for 30’. For cold competition and antibody supershifts, cell extracts were preincubated with the competitor or antibody for 30 min on ice before addition of radiolabeled probe. Competitor double-stranded oligonucleotides were added at a 1000-fold molar excess. Antibodies used were NF-YA Antibody (G-2): sc-17753, and NF-YB Antibody (G-2): sc-376546 (both from Santacruz). The reactions were loaded on 4% polyacrylamide gels and run in 0.5X TBE at 200 V for 2 h.

#### MNase hypersensitivity and Nucleosome Occupancy

Nuclei were prepared from spleen cells from CCAATwt and CCAATm expresser and non-expresser mice and used for MNase digestions as described previously (Kotekar et al. 2008). Briefly, 15X10^6 nuclei were digested for 5’ at 37°C with 0, 3U or 5U of micrococcal nuclease in 100ul of MNase digestion buffer with 1mM CaCl_2_. Nucleosome occupancy was assessed across the PD1 gene (-614, -30, Exon 1 and Exon 5; primers below) by real-time PCR, measured as amplification from 100ng digested DNA (3U MNase) normalized to undigested (0U) DNA.

Additionally, nuclei were digested with high MNase concentrations 50U and 100U for 5’ and 15’ at 37°C to assess accessibility at the PD1 promoter (CCAAT box) by amplifying DNA from the digested soluble chromatin fraction versus the digestion resistant pellet fraction.

**Table:** 

REGION	DIR	SEQUENCE
-640	FWD REV	5’ GGGGCTTTTACATTTCATAGATG 5’ TTTACATTTTACTCTATGGCAAGTCTC
-30	FWD REV	5’ CTTCTCTCTCCTATTGCGTGTCC 5’ ATGATCCTCAGCCTCGGAGT
Exon 1	FWD REV	5’ ACTCCGAGGCTGAGGATCA 5’ CCGCAGCGGCCTTGTTC
Exon 5	FWD REV	5’ CAGACCCTGCTCAGCCCCCCGTCCCCA 5’ ACCTGAGCGCGTCTTCCTCCAGATCACAA

#### Real-time RT-PCR

RNA was prepared using RNeasy kit (Qiagen) according to manufacturer instructions. Each sample was subjected to DNase treatment on column using RNAse free DNase (Qiagen) per manufacturer’s instructions. cDNA was synthesized using oligo dT or random primers and the Superscript III kit according to manufacturer’s instructions (ThermoFisher). Real time PCR was performed as described (Kotekar et al. 2008) using ABI7900 with SYBR PCR Master Mix (Life technologies). For tissue RNA expression, the calculations used the standard curve method, and normalized to the level of 18S in the tissues. All results reported are the average of 2–3 independent experiments in lines derived from different founders, to exclude possible insertion position effects. Endogenous MHC class I levels were determined the same way using published primers (Landel et al. 1997).

#### Assessment of upstream transcription

Tissues were harvested from CCAATwt and CCAATm expresser and non-expresser mice and stored at -80 degrees C. RNA was prepared by using TRIzol (Invitrogen/Thermo Fisher Scientific). DNA contamination was eliminated using TURBO™ DNA-free kit (Invitrogen/Thermo Fisher Scientific). The RNA was then used for cDNA preparation using AffinityScript Multiple Temperature cDNA Synthesis Kit (Agilent) with random primers, oligodT primers or gene-specific primers (Integrated DNA Technologies).

Gene-specific cDNA primers used were:

**Table:** 

Region	For Sense transcripts	For Antisense transcripts
TSE	5’ CTGTCTGGCTCATGGGAAAAC	5’ ACTGATTCAGGTCCACATTCA
USF	5’ TGGAGCCTGAGACCCTGA	5’ CTCACTAAAAGGTTTGGAAATCGC
Promoter proximal	5’ GGGTGGGTGGAGAGTTT	5’ CCCGTGTCCCCAGTTCACTTCTCCG

cDNAs were then amplified using SYBR green PCR master mix (Applied Biosystems,). Primers used for real time RT-PCR were:

**Table:** 

REGION	DIR	SEQUENCE
Exon 2–3	FWD REV	5’ GGAATGTCAAGGAAACCGCAC 5’ ACATGCTCTGGAGGGTGTGAGAC
TSE	FWD REV	5’ TGTGCGGGGCTTTTACATTTC 5’ CACTGGAGGTTTATGTCTGCTTCTG
USF	FWD REV	5’ CACGTGAGGCACTGGAGAC 5’ CCCTGCTGCTCTTCAGAAAGC
Promoter proximal	FWD REV	5’ CGCAACCTGTGTGGGAC 5’ GGGAACCGCAGTGGC

Primers for 18S RNA (AM1716, Ambion/Applied Biosystems) were used for amplifying the internal control. Results were normalized for 18S and copy number.

#### NF-Y siRNA treatment and effect on PD1 expression

93B2 cells (Mouse L-cells stably transfected with the PD1 gene) were cultured for 24-h in 12-well plates and then transfected in duplicate with NF-Y siRNA (Mm_Nfya_3 FlexiTube siRNA, or Mm_Nfya_2 FlexiTube siRNA, or Mm_Nfyb_4 FlexiTube siRNA; Qiagen) using Lipofectamine RNAiMax Reagent (Thermofisher). GAPDH siRNA (Silencer™ Select GAPDH Positive Control siRNA; Thermofisher) and a non-targeting siRNA pool (ON-TARGETplus Non-targeting Control Pool; Horizon/Perkin Elmer) were used as controls.

The cells were harvested at 24, 48 and 72 h. RNA was extracted using RNAeasy plus mini kit. cDNA was prepared using AffinityScript Multiple Temperature cDNA Synthesis Kit (Agilent) with random primers and amplified using SYBR green PCR master mix (Applied Biosystems, Foster City, CA).

siRNA knockdown was confirmed by RT-PCR using NF-YA, NF-YB and GAPDH primers, and effect of NF-Y knockdown on PD1 expression was assessed by RT-PCR using PD1 primers. Primers for 18S RNA (AM1716, Ambion/Applied Biosystems) were used for amplifying the internal control.

Primers used for real time RT-PCR were:

**Table:** 

Primers for:	DIR	SEQUENCE
NF-YA	FWD REV	5’ GGCACAATTCTCCAGCAAG 5’ GGCTCCTGTCTGAACGATCT
NF-YB	FWD REV	5’ AGGGCTGCATTGGAGGTTAAAA 5’ TCCTCAGTATCATCATGGGGC
GAPDH	FWD REV	5’ TGTGTCCGTCGTGGATCTGA 5’ CCTGCTTCACCACCTTCTTGA
PD1	FWD REV	5’ GGAATGTCAAGGAAACCGCAC 5’ ACATGCTCTGGAGGGTGTGAGAC

#### Chromatin Immunoprecipitation (ChIP)

Spleens from 2 transgenic mice were pooled for each experiment. The lysis of the tissues was performed by the MAGNA ChIP Kit for tissues (Millipore). ChIPs were performed according to manufacturer instructions. All experiments were repeated 3 times. Antibodies used: anti Pol-II (Active Motif, Santa Cruz), anti NF-YB (Santa Cruz Biotechnology), anti AcH3 (Abcam), anti Smc1 (Millipore), anti CTCF (Millipore, Santa Cruz), anti H3K4me3, H3K9me3 (Upstate).

#### Quantitation of ChIP results

DNA immunoprecipitated in ChIP reactions was analyzed by real time PCR using the primers described below. (Real-time PCR, rather than ChIP-seq, was used since the analyses focused a transgene.) Quantitative real-time PCR was performed using ABI7900 with SYBR green real time PCR kit (Applied Biosystems). Results were calculated as percentage bound/total input DNA, relative to IgG control.

**Table:** 

Region	DIR	SEQUENCE
-1018	FWD REV	5’ TCCTAATTACCATTCTTCAATCCA 5’ GCTCACAGAATGATTTTCCTTG
-918	FWD REV	5’ CAAGGAAAATCATTCTGTGAGC 5’ GTAAGAGTTTTAAGACCGAATACATTG
-700	FWD REV	5’ ACTGATTCAGGTCCACATTCA 5’ GAGTCCTTTTGGTGGCTGACATC
-640	FWD REV	5’ TGTGCGGGGCTTTTACATTTC 5 ‘ CACTGGAGGTTTATGTCTGCTTCTG
-614	FWD REV	5’ GGGGCTTTTACATTTCATAGATG 5’ TTTACATTTTACTCTATGGCAAGTCTC
-450	FWD REV	5’ CATATGAAATGCATGG 5’ CGTCAGTGGATATTTTCTATACTAG
-400	FWD REV	5’ CTAGTATAGAAAATATCCACTGACG 5’ CCTACAGTTTTACAAATTAGTGAAGACC
-368	FWD REV	5’ CTAGTATAGAAAATATCCACTGACGTATCAACACA 5’ GATTTCCAAACCTTTTAGTGAGAATA
-300	FWD REV	5’ CTCACTAAAAGGTTTGGAAATCGC 5’ CCCTGCTGCTCTTCAGAAAGC
-150	FWD REV	5’ TCAGGGTCTCAGGCTCCA 5’ GGACACGCAATAGGAGAGAGAAG
-75	FWD REV	5’ CGCAACCTGTGTGGGAC 5’ GGGTGGGTGGAGAGTTT
-30	FWD REV	5’ CTTCTCTCTCCTATTGCGTGTCC 5’ ATGATCCTCAGCCTCGGAGT
EXON 1	FWD REV	5’ ACTCCGAGGCTGAGGATCA 5’ CCGCAGCGGCCTTGTTC
EXON 2	FWD REV	5’ GTCCCCACTCCCTGAGCTATTTCTACA 5’ CCACTCCGTAAGTCTGTGCGGTTT
EXON 5	FWD REV	5’ CAGACCCTGCTCAGCCCCCCGTCCCCA 5’ ACCTGAGCGCGTCTTCCTCCAGATCACAA
3’UTR	FWD REV	5’ TGAGAACCTTCCAGAATCCACAT 5’ TCCGTGAAGGGACAAGGACAA

#### Spleen Chromosome conformation capture 3C Analysis

3C analysis was performed as described (Splinter et al. 2004; Cope and Fraser 2009), using Nla III [NEB special order] as the restriction enzyme. Single cell suspensions of ACK treated spleen cells were crosslinked at RT for 5 min with 1% formaldehyde in HBSS/2%FBS, then quenched with glycine to 0.125M for 15min on ice. Washed crosslinked cells were lysed by swelling cold and harvested by rapid centrifugation to obtain nuclei. 2 × 10^7^ cell equivalents were used for each experimental point. 4000 Units of NlaIII [custom concentration 50,000Units/ml] was added and samples were incubated rotating overnight at 37°C. The following morning, enzyme was inactivated with SDS and ligations [T4 DNA ligase, NEB] of varied concentrations were set up for 4 h at 16°C. Samples were RNAse treated, phenol and IAC extracted, and precipitated. PCRs were performed to determine ligated sites. Non-digested, and digested non-ligated samples were used as controls. PCR analysis of 3C experiments were performed using primers in table below.

**Table:** 

Enzyme sites	DIR	SEQUENCE
-689/ + 3434 Enh-Sil/3’UTR	FWD REV	5’ TCCTAATTACCATTCTTCAATCCA 5’ TGGATTCTGGAAGGTTCTCAATC
-689/ + 3434 Enh-Sil/Exon3	FWD REV	5’ TTGTGATCTGGAGGAAGACGCGCTCAGGT 5’ CACTGGAGGTTTATGTCTGCTTCTG
-593/ + 1049 Enh-Sil/Exon3	FWD REV	5’ GGAATGTCAAGGAAACCGCAC 5’ GCGATTTCCAAACCTTTTAGTGA
-488/ + 929 -488/Exon3	FWD REV	5’ GGAATGTCAAGGAAACCGCAC 5’ GCGATTTCCAAACCTTTTAGTGA
+ 35/ + 3040 Exon1/Intron7	FWD REV	5’ CTTCTCTCTCCTATTGCGTGTCC 5’ TGGATTCTGGAAGGTTCTCAATC
+ 1049/ + 2027 Exon3/Exon4	FWD REV	5’ GGAATGTCAAGGAAACCGCAC 5’ TGGGGACGGGGGGCTGAGCAGGGTCTG
+ 2036/ + 2689 Exon4/Intron5	FWD REV	5’ TTGTGATCTGGAGGAAGACGCGCTCAGGT 5’ TGGGGACGGGGGGCTGAGCAGGGTCTG
+ 2717/ + 3302 Intron5/3’UTR	FWD REV	5’ TTGTGATCTGGAGGAAGACGCGCTCAGGT 5’ TGGATTCTGGAAGGTTCTCAATC

#### Methylation assay

Methylation of genomic DNA was examined using methylation sensitive enzymes Aci 1 or Tai 1. 2 ugms of genomic DNA from spleens of CCAATwt and CCAATm mutant mice, were digested with 20 units of either enzyme overnight. PCRs were then performed using oligos flanking the enzymatic sites on the digested DNA and compared to undigested.

**Table:** 

Enzyme sites	DIR	SEQUENCE OF FLANKING OLIGOS
**Aci 1**		
-208, -197	FWD REV	5’ TCAGGGTCTCAGGCTCCA 5’ GGACACGCAATAGGAGAGAGAAG
-41	FWD REV	5’ CTTCTCTCTCCTATTGCGTGTCC 5’ ATGATCCTCAGCCTCGGAGT
+ 92, + 145	FWD REV	5’ GTCCCCACTCCCTGAGCTATTTCTACA 5’ CCACTCCGTAAGTCTGTGCGGTTT
+ 402 to + 602	FWD REV	5’ CGCAACCTGTGTGGGAC 5’ CCGCAGCGGCCTTGTTC
+ 3335	FWD REV	5’ TCTGTGTTCCTATGAGCATCCT 5’ TGGATTCTGGAAGGTTCTCAATC
**Tai 1**		
-414, -309	FWD REV	5’ GCTGAGAACCTGGTCCCCAACTGGAAGAAT 5’ CCCTGCTGCTCTTCAGAAAGC
+ 493	FWD REV	5’ GTCCCCACTCCCTGAGCTATTTCTACA 5’ CCACTCCGTAAGTCTGTGCGGTTT
+ 2393, + 2439	FWD REV	5’ TTGTGATCTGGAGGAAGACGCGCTCAGGT 5’ GCTGAGAACCTGGTCCCCAACTGGAAGAAT
+ 3268	FWD REV	5’ TCTGTGTTCCTATGAGCATCCT 5’ TGGATTCTGGAAGGTTCTCAATC

### Supplementary Information

Below is the link to the electronic supplementary material.

Supplementary file1 (DOCX 1702 KB)

## Data Availability

All materials and data described in this paper are freely available by contacting D. Singer.
