# Supplementary material for: CCAAT Promoter element regulates transgenerational expression of the MHC class I gene
Source: Chromosoma. 2024 Jun 26;133(3):203–16. doi: 10.1007/s00412-024-00820-2 (PMC11266202; doi:10.1007/s00412-024-00820-2)
Supplement: Supplementary file 1 — Supplementary file1 (DOCX 1702 KB) [file 412_2024_820_MOESM1_ESM.docx]

Chromosoma

CCAAT Promoter Element Regulates Transgenerational Expression of the MHC class I Gene

Jocelyn D. Weissman^*^, Aparna Kotekar^*,1^, Zohar Barbash^2^, Jie Mu, Dinah S. Singer^#^

Experimental Immunology Branch, Center for Cancer Research, National Cancer Institute, NIH, Bethesda, MD 20892

# ^1^Current Address: NIH Center for Human Immunology, Inflammation, and Autoimmunity (CHI), National Institute of Allergy and Infectious Diseases, NIH, Bethesda, MD 20892

^2^Current address: FORE biotherapeutics, Philadelphia, PA.

*Co-first authors

#Corresponding author

Dinah S. Singer

Bldg 10, Room 4B-36

NIH, Bethesda, MD 20892

Dinah.Singer@nih.gov

ORCID: 0000-0002-7369-3018

**
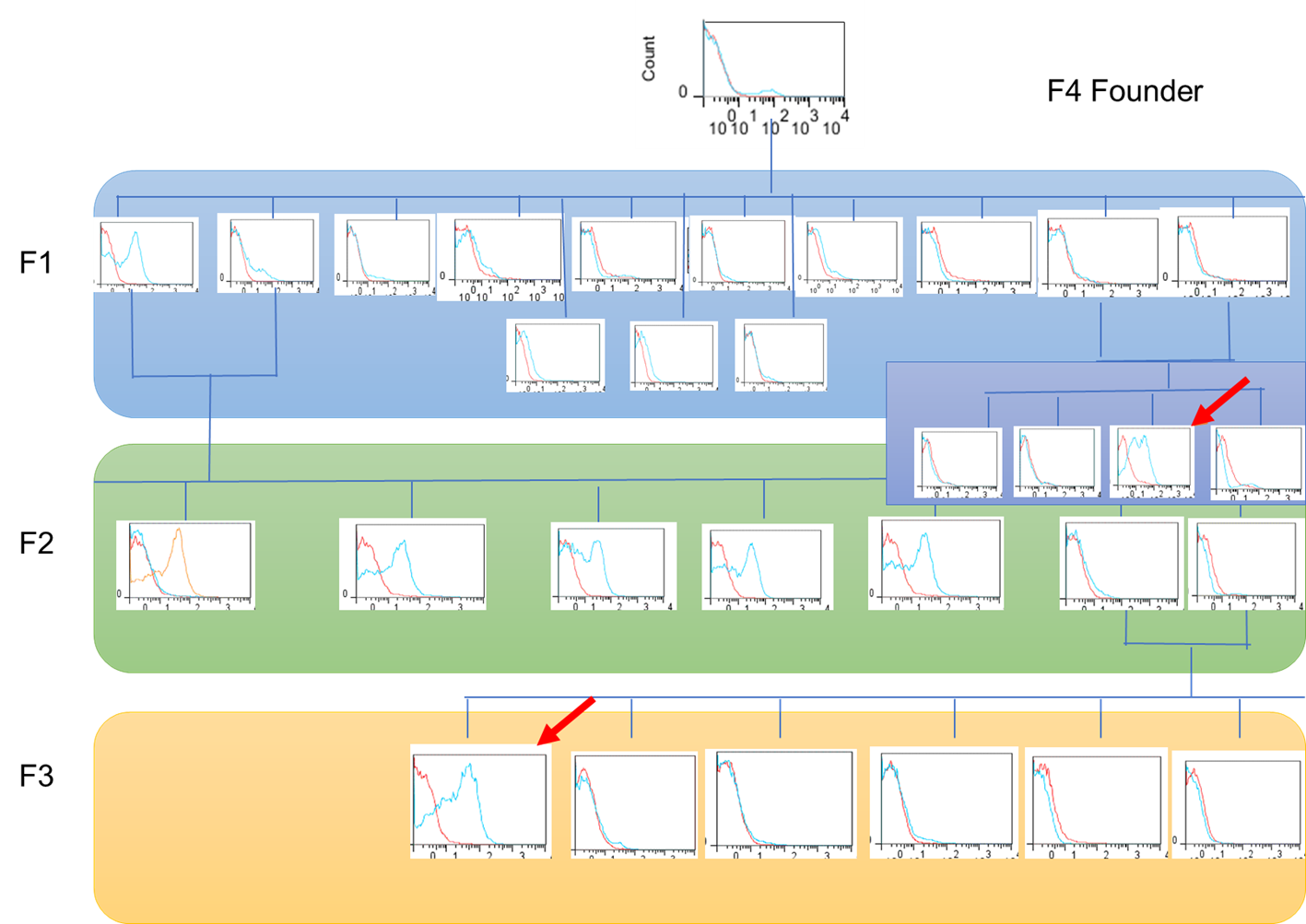
**

**Fig. 1S: Genealogy of the variegated PD1 expression of a CCAATm founder transgenic line, F4, across three generations.**

PBL from the F4 founder and offspring were monitored by FACS for expression. Red arrows highlight examples of offspring that expressed PD1 that were derived from parental non-expressers.

**
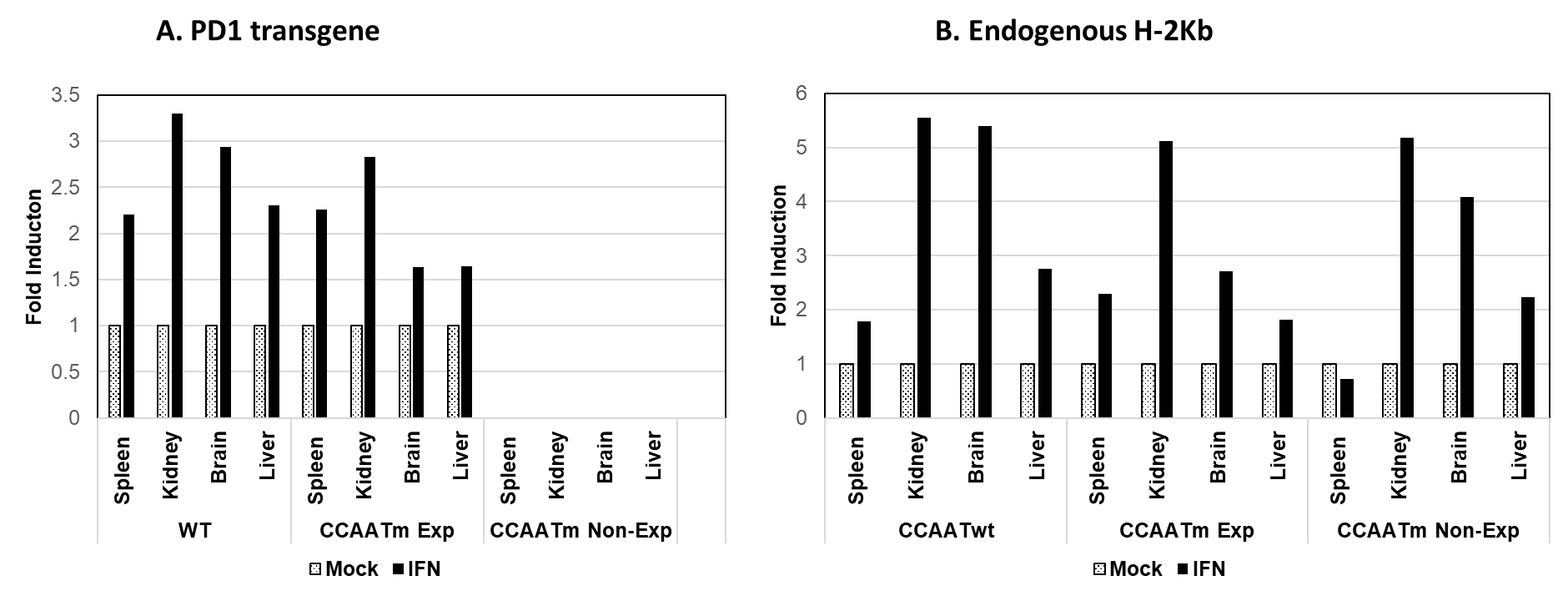
**

**Fig. 2S: CCAATm Expressers, but not Non-expressers, respond to γ-Interferon treatment**

Mice were mock-treated or treated with γ-Interferon; tissues were harvested 24 hours post treatment. Stipple boxes: Mock treated. Black boxes: γ-Interferon treated. A: PD1 RNA levels. B: H2K^b^ RNA levels. Data are presented relative to the mock-treated control and normalized to 18S. Results are representative of two independent experiments.

**
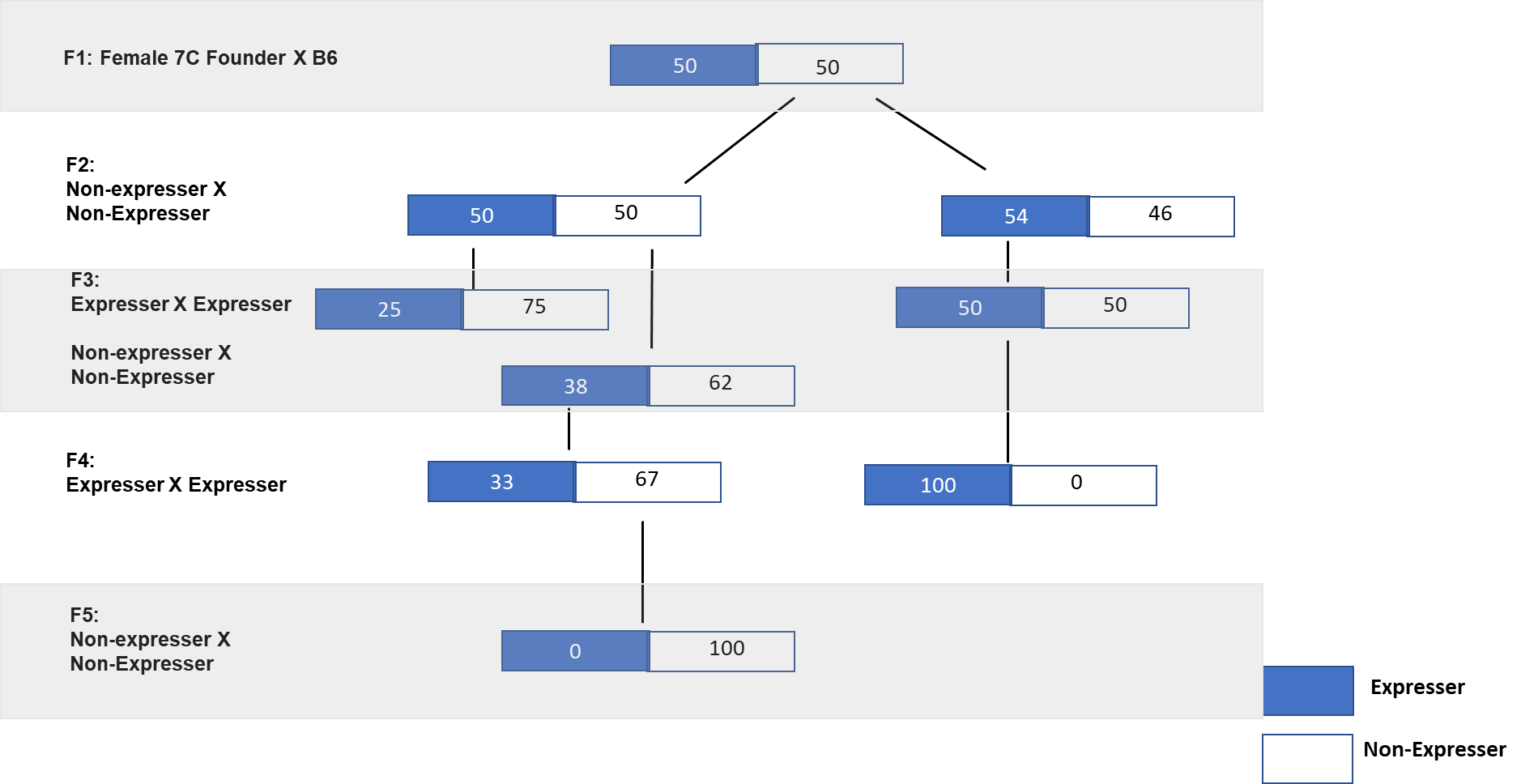
**

**Fig. 3S: Transgenerational epigenetic inheritance in an independent series of transgenic mice**

Variegated expression of MHC class I, PD1, across multiple generations of an independent transgenic mouse line (7C) with a mutated CCAAT core promoter element and a copy number of 24. Transgene-positive off-spring were analyzed by FACS for cell surface PD1 expression on PBL. Only transgene-positive mice that express (solid boxes) or do not express (outlined, white boxes) are shown.


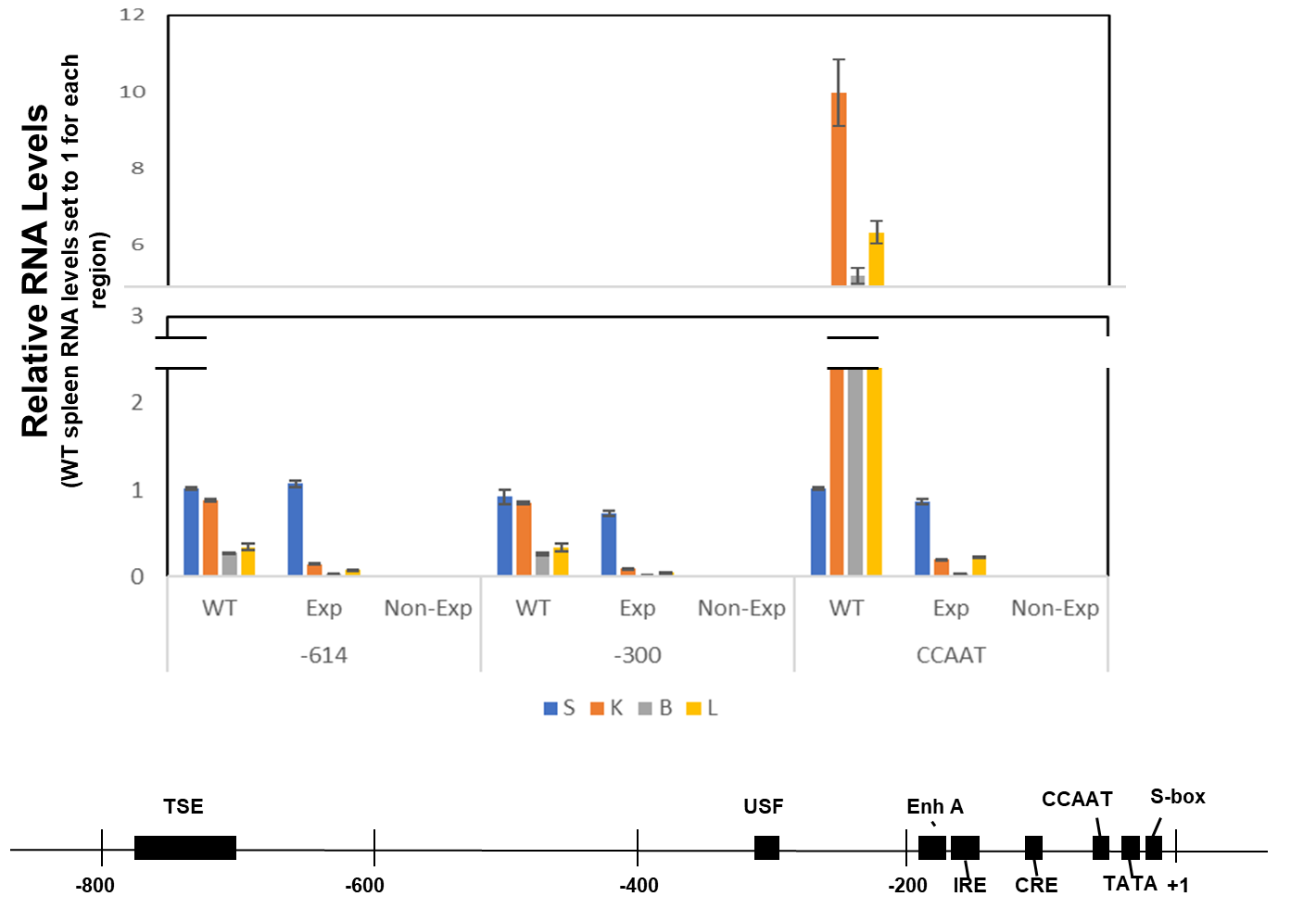


**Fig 4S: Upstream Regulatory Regions of the PD1 Gene are Transcribed in CCAAT Mutant Expressers, but not Non-Expressers**

RNA levels at upstream start sites are shown in tissues from CCAAT mutant expresser and non-expresser mouse strains. CCAATwt spleen RNA levels are set to 1 for each region. Location of upstream PCR primers relative to +1 start site are indicated under the X-Axis (“CCAAT” corresponds to the location of the CCAAT box that is at -30). The data are derived from 2 independent experiments.

**
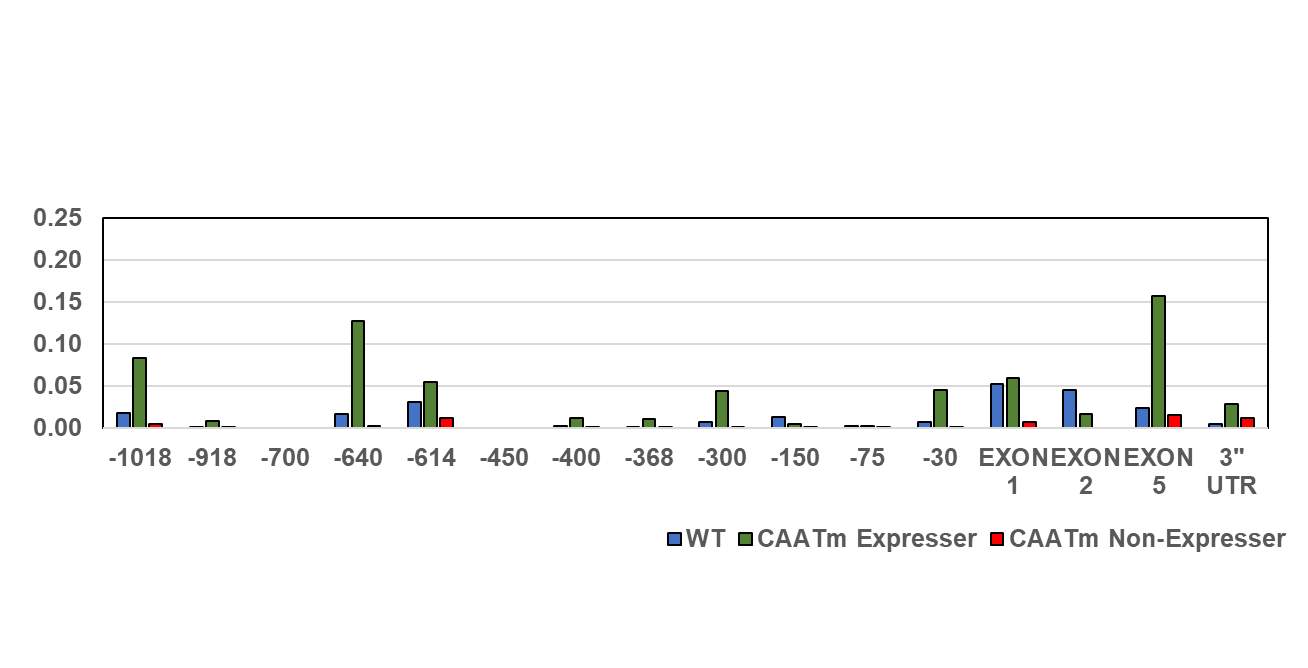
**

**Fig. 5S: H3K27 Methylation patterns do not distinguish CCAATm Expressers from Non-Expressers**

ChIP analysis of H3K27me3 binding to chromatin across the transgene from spleens of CCAATwt, CCAATm expresser and CCAATm non-expresser mice as % of total Input. Note X axis denotes location relative to the TSS and is not to scale. The data are from a single experiment.

**
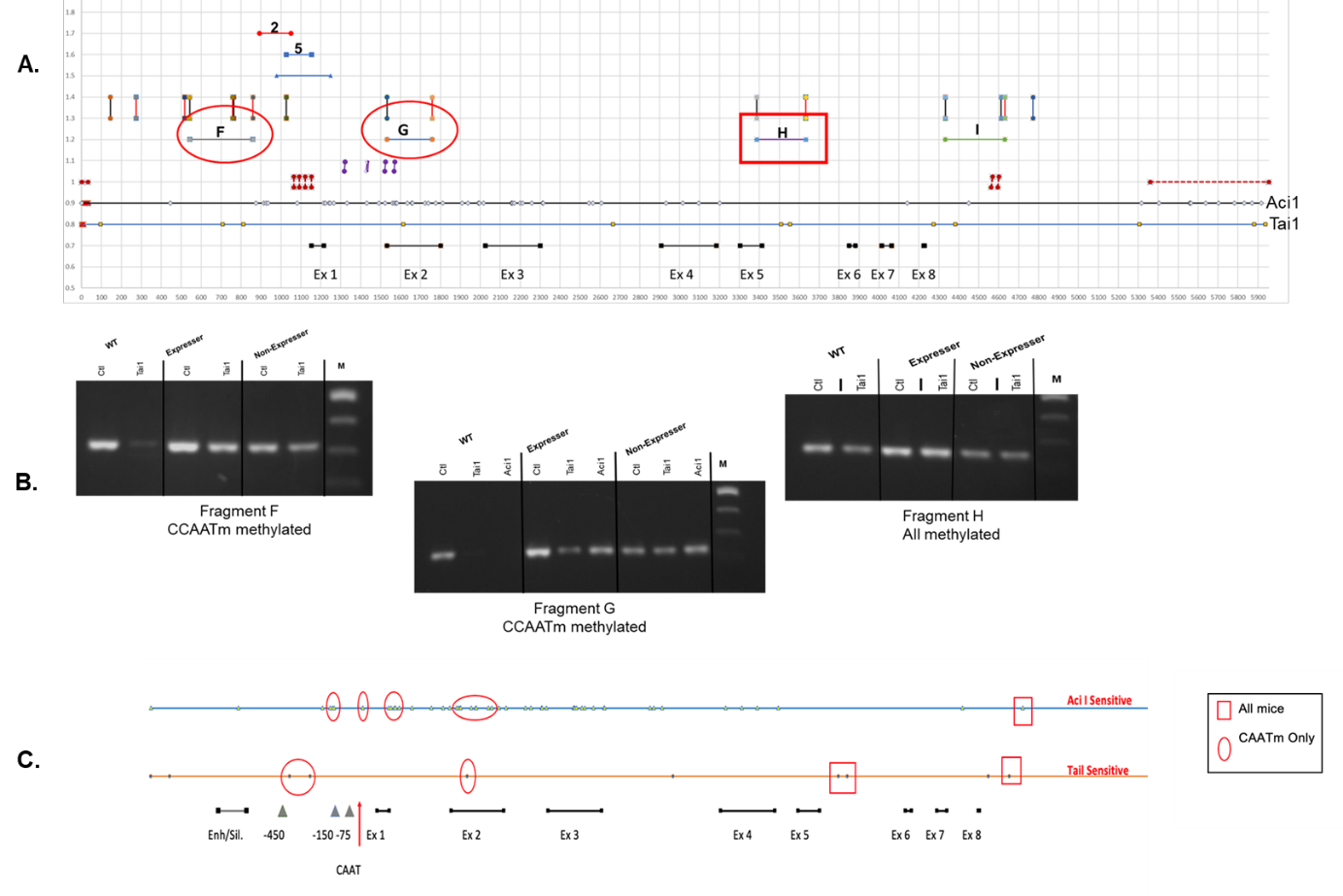
**

**Figure 6S. DNA methylation patterns do not distinguish CCAATm Expressers from Non-Expressers**

A. DNA methylation analysis across the transgene of CCAATwt, CCAATm expresser and CCAATm non-expresser mice. Upper: locations of PCR primers and fragments spanning restriction sites used to assess methylation status. These PCR primers were designed to assay multiple sites across the gene. Representative results, from PCR fragments circled in red, are shown in B. Lower: Restriction enzyme sites for methylation sensitive enzymes Aci 1 (dots, upper line) and Tai1 (squares, lower line) are indicated. Below is a schematic indicating the location of upstream regulatory elements and exons relative to the enzyme sites.

**B.** Representative PCR results for CCAATwt, CCAATm expresser and CCAATm non-expresser mice at restriction sites identified by fragments F, G and H. At Fragments F and G, both CCAATm transgenes, but not the WT are methylated; at Fragment H, all three transgenes are methylated at the site. All experiments were done at least twice.

**C.** Summary of DNA methylation patterns across CCAATwt, CCAATm expresser and CCAATm non-expresser mice. Open oval symbols represent locations of DNA methylation in the CCAAT mutant strains. Open boxes represent locations of DNA methylation in all strains. Below is schematic indicating the location of upstream regulatory elements and exons relative to the enzyme sites.

**
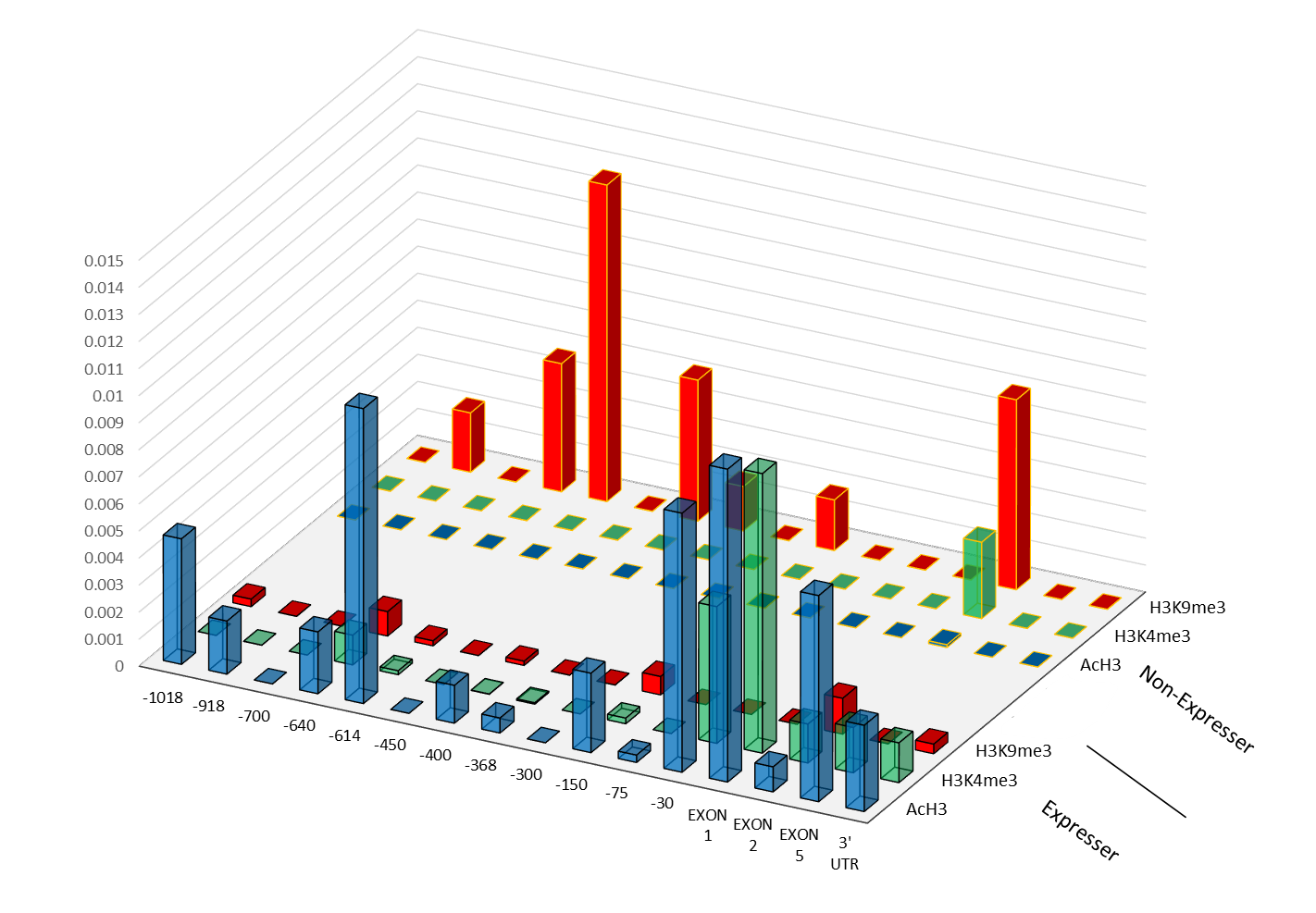
**

**Fig 7S: Histone marks associated with CCAATm transgenes in lines derived from a single independent CCAATm founder correlates with expression.**

ChIP analysis of AcH3, H3K4me3, H3K9me binding to chromatin from spleens of CCAATm expresser and CCAATm non-expresser transgenics (line 7C) which share a common founder, Results are expressed as % of total Input Note: X axis denotes location relative to the TSS and is not to scale. The results are representative of two independent experiments.

**
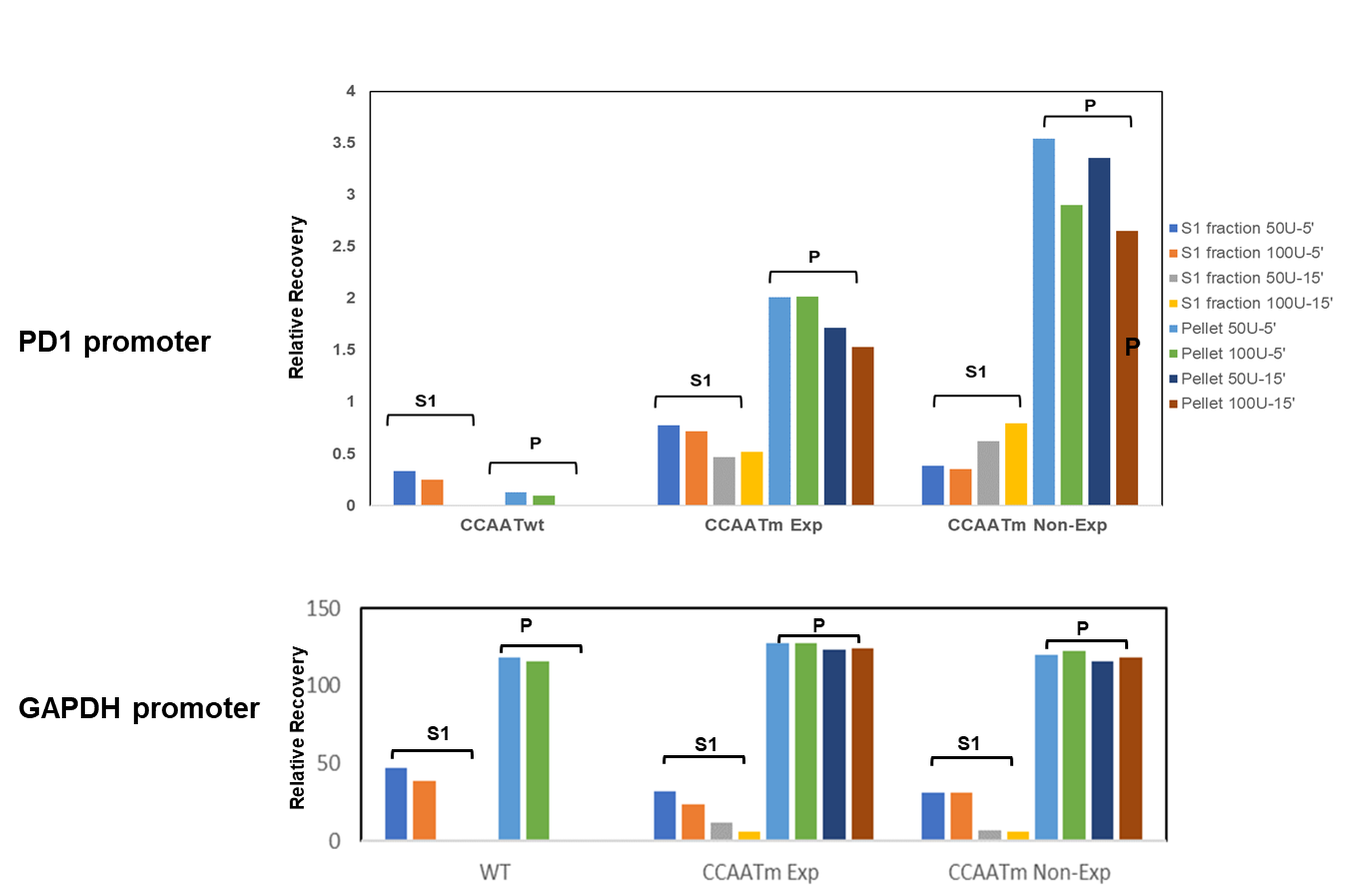
**

**Figure 8S: PD1 Promoter DNA is relatively inaccessible in CCAAT Mutants relative to CCAATwt**

Nuclei from spleens of CCAATwt, CCAATm expresser and CCAATm non-expresser mice were digested with 50 or 100 units of MNase and recovery of DNA in the supernatants (MNase-sensitive fraction) and pellets (MNase-resistant fraction) were assessed by PCR for recovery of the PD1 promoter (upper panel) or GAPDH (lower panel).

S, K, B and L represent data from spleen, kidney, brain and liver, respectively.

**
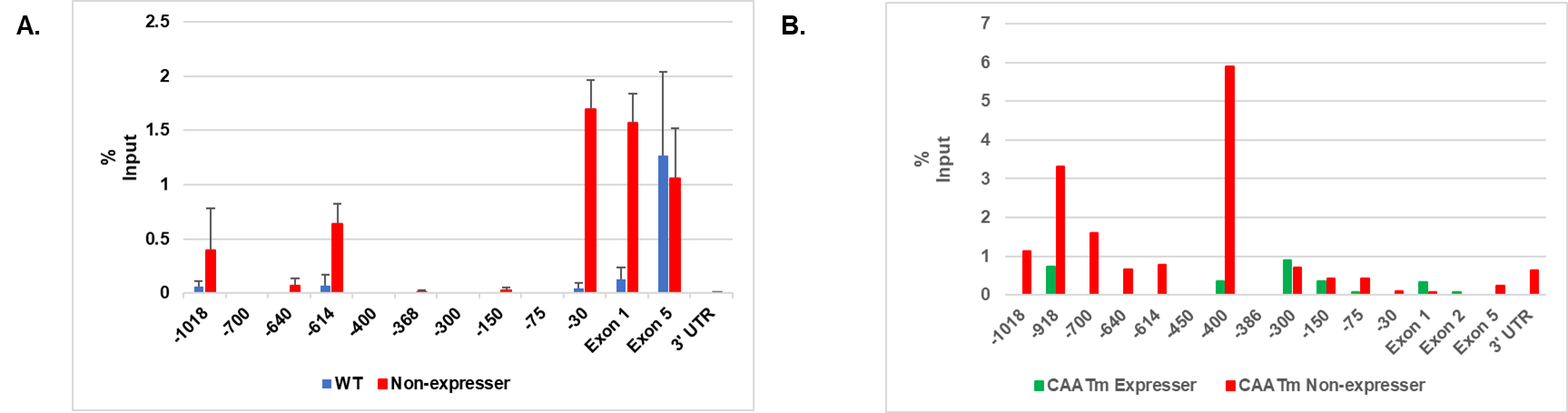
**

**Fig. 9S: The patterns of CTCF and cohesin binding to the PD1 gene in CCAATwt, CCAATm Expressers and non-Expresser mice**

A. ChIP analysis of CTCF binding to chromatin from spleens of CCAATwt and CCAATm non-expresser transgenic strains as % of total Input. Data represent average of two biological replicates.

B. ChIP analysis of CTCF binding to chromatin from spleens of CCAATm expresser and CCAATm non-expresser transgenic strains as % of total Input.

Note X axis denotes location relative to the TSS and is not to scale. Results are representative of 3 experiments.


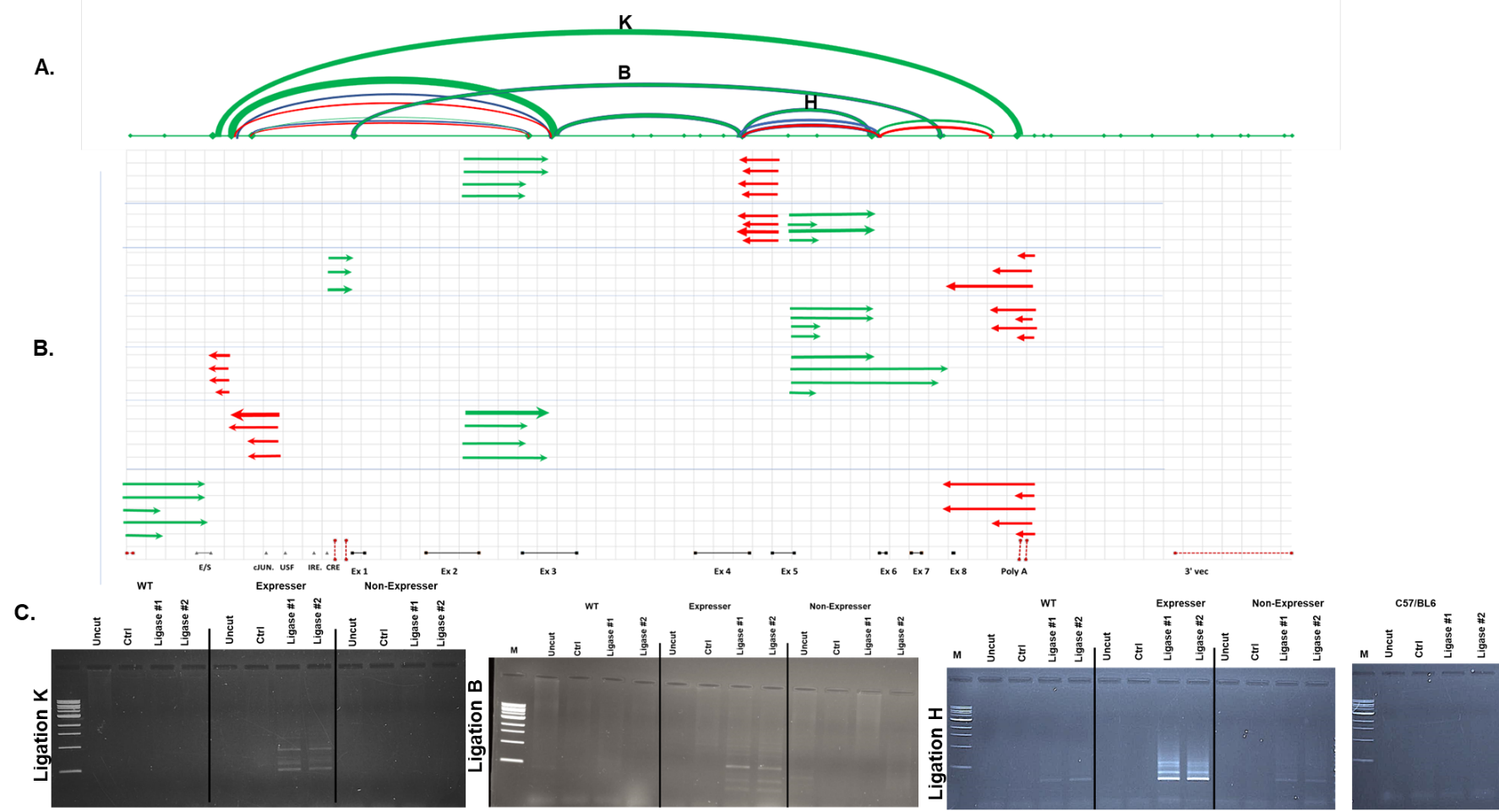


**Fig. 10S 3C:** **Looping patterns distinguish the CCAATm Expressers from the WT and Non-Expressers.** CCAATm expresser transgenes generate DNA loops not seen in either CCAATwt or the CCAATm non-expressers transgenes.

1. The endpoints for each loop are located at sites of enzymatic digestion which were ligated as seen by flanking PCRs using the oligos listed in Methods. The data are representative of 3 independent experiments. The thickness of the loop correlates with intensity of the PCR bands.
2. The location of the different primers used to map the loops. The positions of the exons and upstream regulatory regions are indicated at the bottom.
3. Three representative ligations, spanning loops K, B and H (as shown in A.) are shown for the WT, CCAATm expresser and CCAATm non-expresser spleen. For each sample, lane 1 control without NaiI; lane 2, control NaiI digest without subsequent ligation; lanes 3 and 4, two independent NaiI digestions and ligations.
